# Ensemble transformer-based multiple instance learning for predicting neoadjuvant chemotherapy response from breast cancer biopsy whole-slide images

**DOI:** 10.3389/fonc.2026.1728511

**Published:** 2026-02-23

**Authors:** Zhenshui Wu, Kaining Ye, Jianming Weng, Zhongping Zhang, Xuehong Liao, Kaixin Du

**Affiliations:** 1 Department of Pathology, The Second Affiliated Hospital of Fujian Medical University, Quanzhou, Fujian, China; 2 Department of Pathology, Zhangzhou Affiliated Hospital of Fujian Medical University, Zhangzhou, Fujian, China; 3 Department of Pathology, Sapporo Medical University, Sapporo, Japan; 4 Department of Radiation Oncology, Fujian Medical University Xiamen Humanity Hospital, Xiamen, Fujian, China

**Keywords:** breast cancer, multiple instance learning, neoadjuvant chemotherapy, pathomics, PCR, transformer

## Abstract

**Introduction:**

Neoadjuvant chemotherapy (NAC) is a cornerstone of breast cancer management, and accurate prediction of therapeutic efficacy is essential for optimizing treatment strategies and improving patient outcomes.

**Methods:**

This study proposes an integrated Transformer-based Multiple Instance Learning (MIL) framework that leverages pre-treatment biopsy whole-slide images (WSIs) to predict NAC response. A multi-institutional dataset of 128 patients was collected, comprising 86 cases for training, 42 for internal validation, and 22 microscope images for external validation. The framework integrates ResNet50 feature extraction, a multi-scale attention Transformer encoder, and a two-stage classification strategy to capture both local morphological and global contextual features. Class imbalance was mitigated using SMOTE and ADASYN, while domain adaptation (DANN) and metric learning enhanced cross-modal robustness.

**Results:**

The proposed model achieved a WSI-level accuracy of 79.3% in internal validation and demonstrated strong discriminative ability in identifying pathological complete response (pCR, AUC = 0.82) and non-response (AUC = 0.77). External validation using lower-resolution microscope images yielded an AUC of 0.70 for pCR and 0.67 for non-response, outperforming traditional CNN architectures such as GoogleNet, ResNet34, and SqueezeNet. The model’s heatmap visualizations revealed well-defined lesion boundaries and interpretable regions of interest, underscoring its clinical transparency.

**Discussion:**

By relying solely on hematoxylin and eosin-stained WSIs, the framework provides a fully automated, interpretable, and resource-efficient approach suitable for real-world deployment. The two-stage classification design offers fine-grained stratification between pCR, partial, and poor responders, which is critical for personalized therapy planning. Future work will focus on expanding multi-center datasets and integrating advanced pathology foundation models to further enhance cross-domain generalization and clinical applicability.

## Simple summary

This study introduces a new deep learning model to predict how well breast cancer patients will respond to neoadjuvant chemotherapy (NAC) using only pre-treatment biopsy whole-slide images (WSIs). The model, based on a Transformer and Multiple Instance Learning (MIL), was tested on 128 patient samples from multiple centers, achieving high accuracy (79.3% for WSIs) in predicting complete response (pCR) and non-response to NAC. It uses a two-stage classification approach to distinguish between good, partial, and poor responses, outperforming traditional models like GoogleNet and ResNet34. All without manual ROI (Region Of Interest) annotation, keeping the pipeline fully automated and pathologist-friendly. The model’s ability to work with single-modal data makes it practical for resource-limited clinics, and its performance was validated externally with microscope images. Future improvements could involve larger datasets and better handling of class imbalances to enhance accuracy and applicability in personalized breast cancer treatment.

## 1 Introduction

Breast cancer remains the most prevalent malignancy among women worldwide, with an estimated 19.3 million new cases and 10.0 million deaths in 2020 (1). Neoadjuvant chemotherapy (NAC) is widely regarded as a cornerstone in the management of locally advanced breast cancer, demonstrating significant efficacy in tumor downstaging, tumor size reduction, and enhancement of breast-conserving surgery rates (2). Pathological complete response (pCR) has been consistently linked to improved event-free survival (EFS) and overall survival (OS), with pCR rates reaching up to 60% in triple-negative (TNBC) and HER2-positive subtypes (3, 4). Despite its benefits, the heterogeneity of tumors often renders some patients unresponsive to NAC, potentially leading to prolonged treatment cycles, adverse side effects, and delays in achieving optimal therapeutic outcomes (5). As such, the accurate prediction of pCR or non-response prior to initiating therapy is imperative to refine treatment strategies.

Pathomics, which involves the analysis of digitized whole-slide images (WSIs), has emerged as a promising approach for predicting treatment response by extracting microscopic features (6, 7). Unlike radiomics, which focuses on macroscopic imaging features, pathomics captures nuanced alterations in the tumor microenvironment, including nuclear atypia and cellular infiltration (8). The recent advent of deep learning techniques, particularly Transformer-based multiple instance learning (MIL), holds significant promise in the processing of WSIs by effectively modeling complex pathological features and enhancing prediction accuracy. MIL, which interprets images as collections of instances (patches), learns the interrelationships between patches to predict overall labels, making it well-suited for the intricacies and heterogeneity inherent in pathological images (9).Recent advances in MIL have further improved WSI classification performance through critical instance selection, hierarchical feature modeling, and iterative feature refinement (10–13).

Pre-treatment biopsy WSIs provide a wealth of histological data critical for predicting treatment responses. While several studies have concentrated on NAC response prediction, the majority focus on radiomics. Pathomics research remains relatively sparse due to the intricate data processing requirements and the need for high-resolution images. Zhang et al. (14) integrated radiomics, pathomics, and clinical data to develop the DLRPM model, which achieved an impressive AUC of 0.927 for pCR prediction. However, the model’s reliance on multimodal data, an SVM-based architecture, single-center data, and semi-automatic segmentation methods presents limitations in terms of generalizability and efficiency. Aswolinskiy et al. (15) reported AUC values ranging from 0.66 to 0.88 for their PROACTING model, applied to TNBC and Luminal B subtypes, though the model’s performance exhibited variability, particularly in lymphocyte detection and mitotic rate (MTR) assessment. Li et al. (16) achieved an AUC of 0.82 with their DLPCM model by combining clinical features and stromal TILs, outperforming the single-modal DLPM (AUC 0.63–0.72). However, the model’s external validation results (AUC 0.70–0.72) were less robust, and it lacked sufficient performance metrics for F1 scores and minority class predictions.

**Figure 1:**
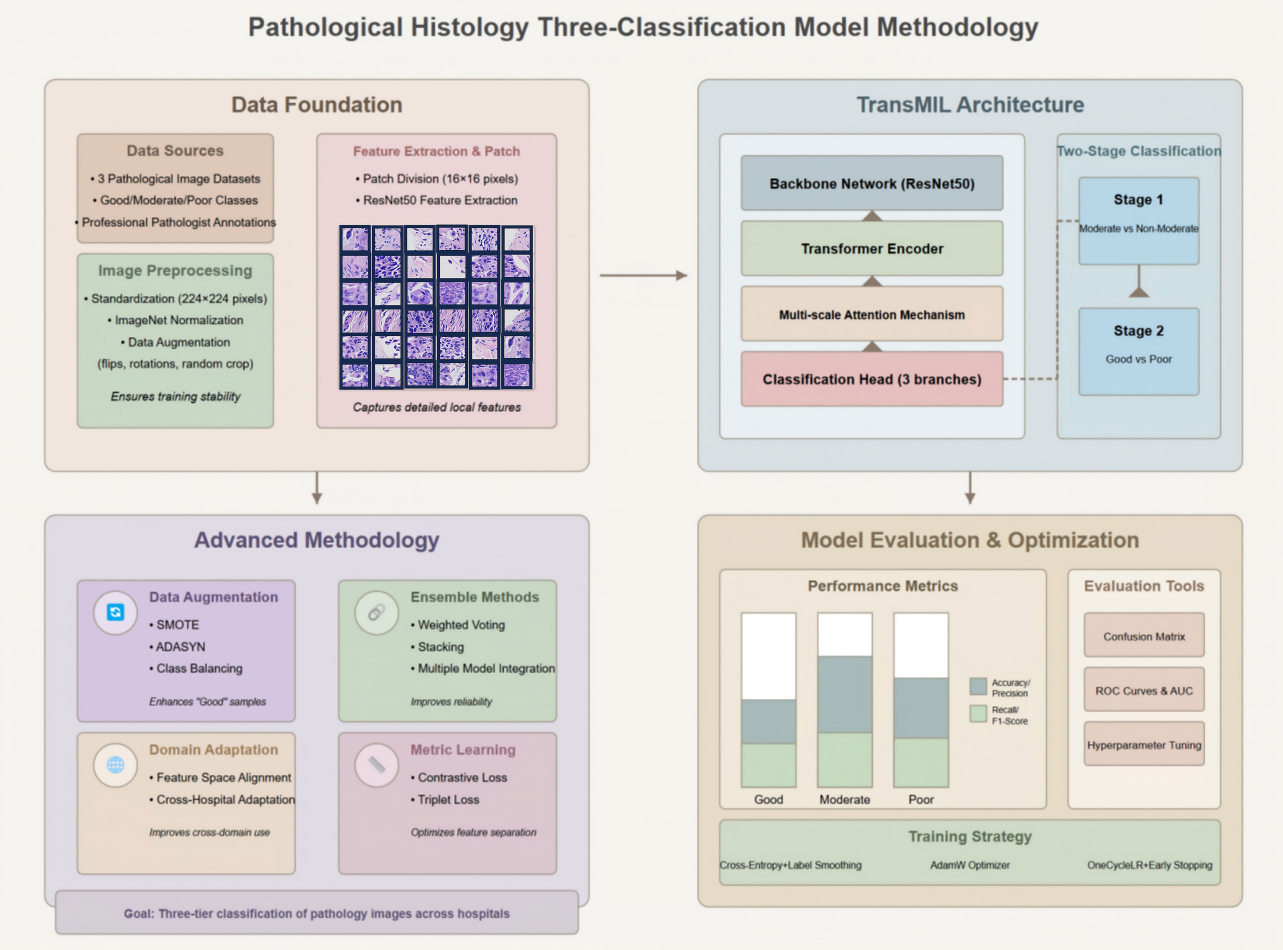
Overview of the pathological three-class classification model.

Current pathomics research tends to focus primarily on binary classification (pCR vs. non-pCR) (14–18), often overlooking patients who are non-responsive or exhibit poor responses to treatment, despite the significant toxicity they may endure from extended chemotherapy. Furthermore, the reliance on multimodal data and manual segmentation impedes the broader applicability and efficiency of these models. In response, this study introduces a novel deep learning model built on the TransMIL framework (**Figure 1**), which has been optimized for feature extraction, data augmentation, and model integration, with a focus on effectively mining critical features from breast cancer pathological images. This approach adopts a single-modal design, utilizing only pre-treatment WSIs, thereby simplifying the complexities associated with multimodal data acquisition and offering superior generalizability, particularly in resource-limited clinical settings. A key innovation of this model is its two-stage classification strategy, which simultaneously identifies patients with pathological complete response (pCR) and those with no or poor response to NAC, delivering clinically meaningful stratified information. This paper details the data preprocessing workflow, model architecture, training and optimization methodologies, and evaluation metrics, while further validating the model’s potential for real-world clinical implementation through external validation.

## 2 Materials and methods

### 2.1 Data collection and preprocessing

#### 2.1.1 Data source

The training dataset consisted of 128 whole slide images (WSIs) from biopsies collected between November 2017 and August 2024 at Zhangzhou Affiliated Hospital, Xiamen Humanity Hospital, and Fujian Medical University Second Hospital. These images were divided into a training set (86 cases) and an internal validation set (42 cases, in an 8:2 ratio). External validation was conducted with 22 microscope images obtained from Fujian Medical University Second Hospital between April 2019 and July 2024. The WSIs were scanned using a Motic VM1000 system (40× magnification, 0.25 μm/pixel), while the microscope images were captured with a Leica DM3000 (20Xor 40Xmagnification, 0.5μm or 0.25μm/pixel). All samples were H&E-stained pre-NAC biopsies, and the study received approval from both the Zhangzhou Hospital Ethics Committee (Approval No.: 2025LWB22) and the Fujian Medical University Second Hospital Ethics Committee (Approval No.: 2025543), in strict compliance with the Helsinki Declaration. Given the retrospective nature of the study, written informed consent from patients was waived.

Using the Miller-Payne grading system, the dataset was classified into three categories: Good (pathologic complete response, pCR, Grade 5, 60 cases), Medium (partial response, Grades 2-4, 51 cases), and Poor (no response, Grade 1, 17 cases). Grading was conducted independently by two pathologists, with any discrepancies resolved by a third pathologist with 15 years of experience. For external validation, microscope images were utilized to simulate clinical scenarios in resource-limited settings, thereby enhancing the model’s real-world applicability.

#### 2.1.2 Image preprocessing

Initially, pathology experts reviewed the whole slide images (WSIs) to eliminate blank or non-tissue regions. Subsequently, tissue regions were delineated, and non-overlapping image patches (256×256 pixels) were extracted from valid tissue areas at 40× magnification. To meet the input requirements of the deep learning architecture, all patches were uniformly resized to 224×224 pixels prior to model input. Image color normalization was applied using ImageNet standard parameters (mean = [0.485, 0.456, 0.406], standard deviation = [0.229, 0.224, 0.225]), aimed at mitigating the impact of staining variability across different slides. Data augmentation techniques, including random horizontal/vertical flipping, ± 30° random rotation, random cropping, and scaling between 0.8 and 1.2 times, were employed to enhance the model’s ability to generalize across morphological variations in tissue. The same preprocessing pipeline was applied to external microscope images, ensuring consistency in input distribution across modalities.

Following tissue segmentation and patch standardization, a pretrained ResNet50 model was utilized to extract features from each patch. Trained on ImageNet, this model captures a wide range of multidimensional features from pathological images, including cellular morphology (e.g., size, nuclear membrane integrity, nuclear-cytoplasmic ratio), tissue structure (e.g., ducts, glands, necrotic areas), texture features (e.g., fibrotic regions, nuclear staining density), and staining properties. Each patch generates a 1024-dimensional feature vector, which is then passed to a Transformer module to model spatial correlations between different regions. The preprocessing workflow for microscope images remained consistent with that of WSIs to ensure uniform input dimensions and structural integrity during the validation phase.

### 2.2 Model architecture and training

#### 2.2.1 TransMIL architecture

The model employed in this study, built upon the TransMIL framework (**Figure 1**), combines ResNet50 and a multi-layer Transformer encoder. ResNet50 is responsible for extracting low-level features, while the Transformer utilizes multi-head self-attention mechanisms to capture long-range dependencies between patches. A multi-scale attention strategy (with scales of 4×4, 8×8, and 16×16 pixels) is implemented to capture both microscopic features (e.g., nuclear atypia) and macroscopic characteristics (e.g., tumor infiltration). The classification head generates predictions of “Good,” “Medium,” and “Bad” through three distinct branches, with the final outcome determined by weighted voting across the branches.

#### 2.2.2 Two-stage classification

A two-stage classification strategy was implemented: in the first stage, a binary classifier distinguishes between “Medium” and “non-Medium” (combining “Good” and “Bad”), while the second stage differentiates “Good” from “Bad.” This approach minimizes class confusion and improves the clarity of decision boundaries. Training was conducted using cross-entropy loss with label smoothing (ϵ = 0.1) to mitigate overfitting.

#### 2.2.3 Training strategy

**Figure 2:**
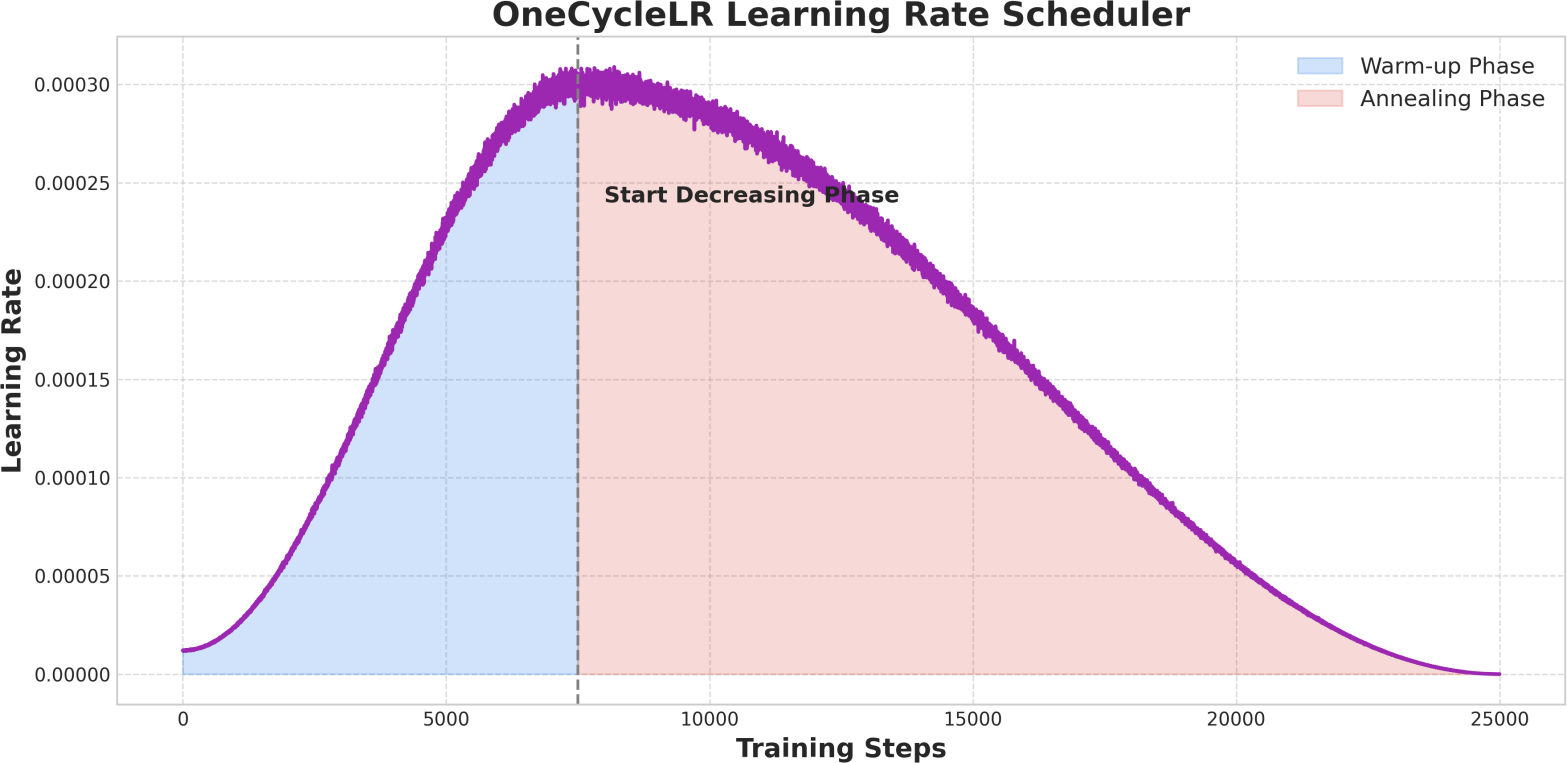
OneCycleLR learning rate trend. The warm-up phase gradually increases the learning rate, the start-decreasing phase reduces it after peaking, and the annealing phase lowers it to near zero.

To enhance model performance and generalization, this study incorporated a comprehensive approach that combined loss function optimization, careful optimizer selection, data augmentation, domain adaptation, and metric learning. Cross-entropy loss with label smoothing (factor = 0.1) addressed the class imbalance in the “Bad” category (17 cases), improving predictions for the minority class. The AdamW optimizer (initial learning rate = 1e-4, weight decay = 0.01) with momentum and regularization facilitated faster convergence, while OneCycleLR scheduling (maximum learning rate = 3e-4, 25,000 steps) enabled dynamic learning rate adjustments-starting with a warm-up phase for stability, peaking for rapid convergence, and annealing for parameter refinement (**Figure 2**). Data augmentation strategies, including random flips, rotations (± 30°), cropping, and scaling (0.8-1.2X), were complemented by synthetic sample generation using SMOTE (k=5 neighbors) and ADASYN for the “Bad” class, reducing overfitting and improving generalization. Domain adversarial training (DANN, discriminator learning rate = 1e-5) aligned the feature spaces of multi-center WSIs and microscope images, thereby boosting cross-domain robustness. Metric learning techniques, such as contrastive loss (Euclidean distance, margin m=1.0) and triplet loss (m=0.5), were employed to optimize patch feature separability, ensuring clear distinctions between the “Good,” “Medium,” and “Bad” categories. Finally, ensemble learning, leveraging five models and weighted voting based on validation F1 scores, further stabilized predictions. Training was conducted with a batch size of 64, early stopping (patience = 10 epochs), and TensorBoard monitoring to ensure efficient convergence and robustness.

#### 2.2.4 Model evaluation and tuning

To thoroughly assess the model’s performance in the three-class classification task, five key metrics were utilized for quantitative analysis: Accuracy, Precision, Recall, F1-score, and Area Under the Curve (AUC). In addition, confusion matrices, Receiver Operating Characteristic (ROC) curves, and Precision-Recall (PR) curves were generated to visually capture the model’s discriminative capability across different classes.

Hyperparameter optimization was performed using a Grid Search approach, where critical parameters, including the initial learning rate (ranging from 1e-5 to 1e-3), batch size (8, 16, 32), and the number of attention heads in the Transformer module, were systematically adjusted. All hyperparameter tuning was conducted on the validation set to ensure optimal performance, thereby avoiding any reliance on the test set results.

### 2.3 Comparison models

To validate the effectiveness and superiority of the proposed model, three representative image classification architectures—GoogleNet, ResNet34, and SqueezeNet—were selected for comparative evaluation. All models were pretrained on the ImageNet dataset and modified to include a three-class output layer tailored to the present task. Each comparative model was trained under identical experimental conditions as the proposed framework, including the same training dataset, labels, optimizer, loss function, and number of training epochs, thereby ensuring a fair and consistent comparison across architectures. Performance evaluation was conducted on both the internal validation and external test datasets to provide a comprehensive assessment of model robustness and generalizability.

### 2.4 Experimental environment and software/hardware configuration

All model training and evaluation in this study were performed on a Linux-based system (Ubuntu 20.04). The computational platform was equipped with an Intel Core i9-10900K CPU, 128 GB of RAM, and a single NVIDIA GeForce RTX 3090 GPU (24 GB VRAM). This configuration provided sufficient computational resources for large-scale pathological image processing and efficient training of the Transformer-based architecture, ensuring timely completion of all experiments. The software environment utilized Python 3.9 as the primary programming language and PyTorch 1.12.1 as the deep learning framework. Image preprocessing and data augmentation were implemented using Torchvision 0.13.1 and OpenCV 4.6. Model evaluation, including metric computation, confusion matrix generation, and grid search optimization, was conducted with scikit-learn 1.1. Visualization of images, training curves, and evaluation results was achieved using matplotlib and seaborn. GPU acceleration was enabled through CUDA 11.3 and cuDNN 8.2, with all experiments executed in a single-GPU mode to maintain consistency and reproducibility.

## 3 Results

### 3.1 Model training process

#### 3.1.1 OneCycleLR learning rate scheduler

**Figure 2** depicts the evolution of the learning rate throughout model training under the OneCycleLR scheduling strategy. Across 25,000 training steps, the learning rate increased from 0 to approximately 0.0003 during the warm-up phase (first 5,000 steps), reached its peak, and then progressively declined during the start-decreasing phase (5,000-10,000 steps), ultimately approaching 0 in the annealing phase (10,000-25,000 steps). This scheduling approach effectively stabilized training dynamics, expedited convergence, and refined parameter optimization, thereby enhancing the model’s overall performance.

#### 3.1.2 Training curve comparison

**Figure 3:**
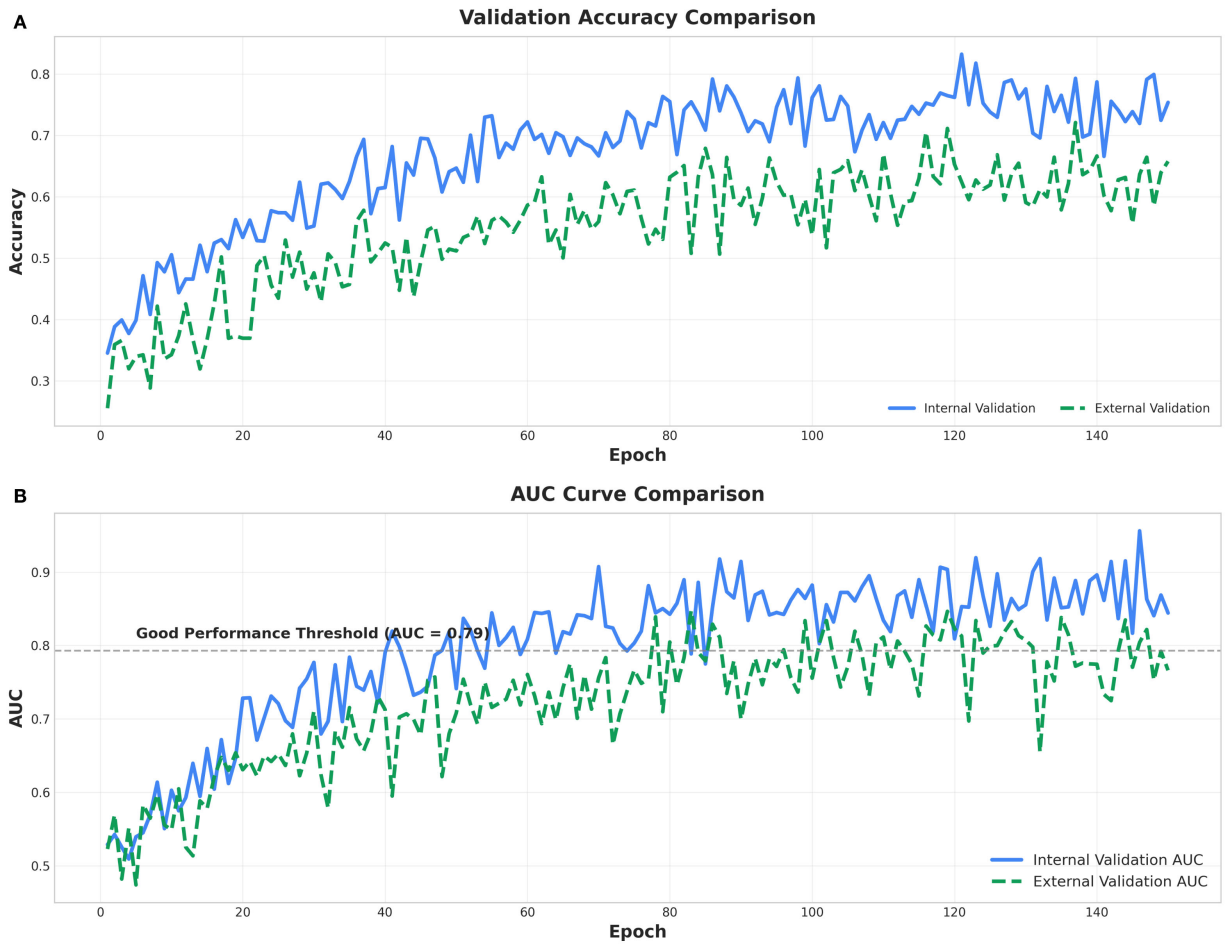
Two line charts **(A, B)** comparing internal and external model performance over 150 epochs. **(A)** Validation Accuracy Comparison. Validation accuracy, where internal validation (blue solid line) remains higher than external validation (green dashed line). **(B)** AUC Curve Comparison. Area under the curve (AUC), with a marked threshold of 0.8; internal validation AUC consistently exceeds external validation AUC. Both charts include legends indicating line styles for internal and external validation.

**Figure 3** presents the accuracy trajectories over 140 training epochs for internal (blue curve) and external (green curve) validation datasets. Overall accuracy increased from approximately 0.40 to the range of 0.70-0.80, exhibiting a steady upward trend despite minor fluctuations. The comparable performance across both validation sets indicates promising generalization capability, although slight discrepancies between the curves suggest residual overfitting. Internal validation accuracy consistently exceeded that of external validation, implying a mild degree of overfitting to the training data. The area under the ROC curve (AUC) for both validations also improved progressively throughout training, converging toward approximately 0.8-approaching the “Good Performance Threshold”. The marginally higher internal AUC values further reflect superior discriminative ability on the training data. Collectively, these findings demonstrate gradual performance enhancement during model training, with internal results outperforming external ones, reinforcing the likelihood of mild overfitting. Nevertheless, an AUC nearing 0.8 denotes strong classification capability, and additional optimization could further improve model robustness. The performance achieved in this study surpasses that of comparable models (see **Supplementary Data Figures S1-1**, **S2-1**, **S3-1**).

**Figure 4:**
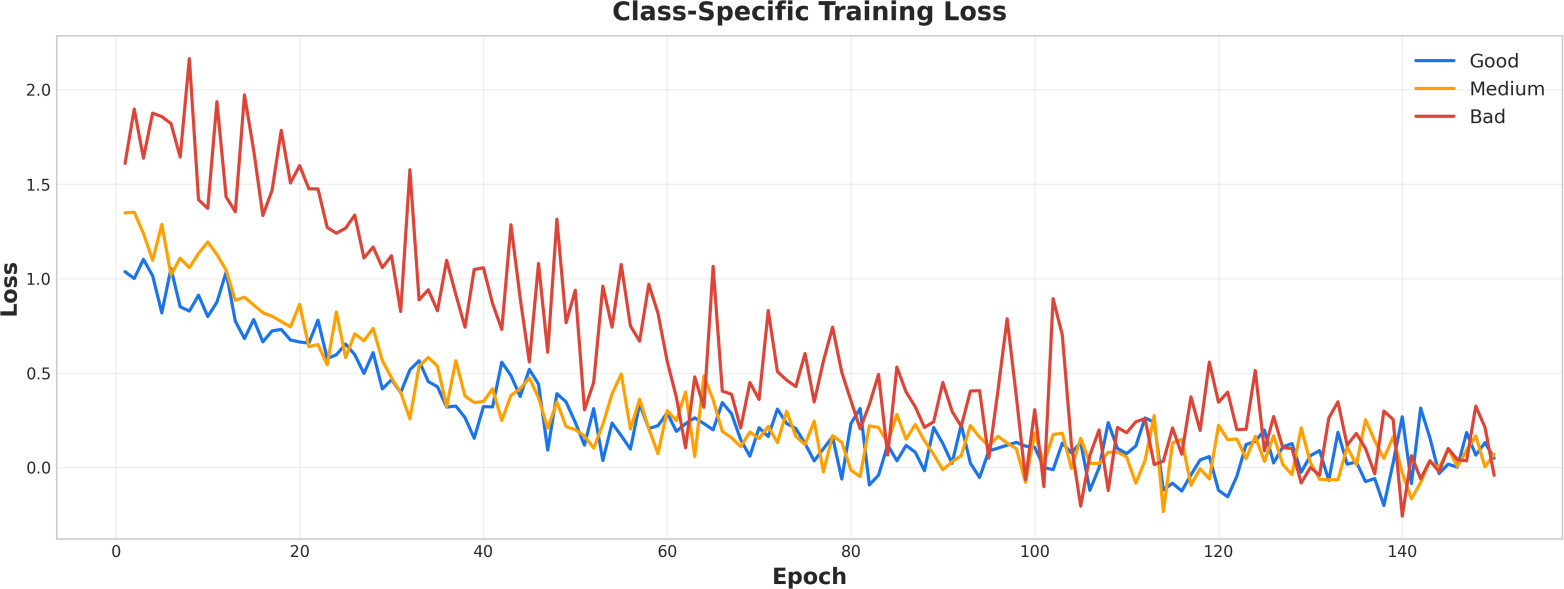
Class-specific training loss curves. The x-axis represents training epochs, and the y-axis represents loss values. Blue, yellow, and red lines indicate complete response, partial response, and non-response, respectively.

**Figure 4** illustrates the class-specific training loss trends over 140 epochs for complete response (Good, blue), partial response (Medium, yellow), and non-response (Bad, red). The initial loss value was approximately 2.0, progressively decreasing with reduced fluctuations, ultimately stabilizing between 0.0 and 0.5 by epoch 140. The losses for complete and partial responses exhibited a smooth decline, while non-response losses demonstrated greater fluctuations, though still following a general downward trajectory. Comparative performance results from alternative models can be found in **Supplementary Data Figures S1-2**, **S2-2**, **S3-2**.

### 3.2 Model performance comparison

#### 3.2.1 Comparison of performance metrics for each model

The model developed in this study was evaluated using key performance metrics, including precision, recall, F1 score, accuracy, and specificity. The F1 score, the harmonic mean of precision and recall, is particularly suitable for assessing models trained on imbalanced datasets. In the internal validation set, the model exhibited excellent performance in predicting complete response (pCR), achieving precision, recall, and F1 scores of 0.85, 0.80, and 0.82, respectively, with accuracy and specificity of 0.74 and 0.75 (**Table 1**). In the external validation set, performance showed a moderate decline, with corresponding scores of 0.70, 0.68, and 0.70, and an accuracy of 0.70 (**Table 1**). These results demonstrate the model’s strong capability in predicting pCR, providing valuable support for clinical decision-making.

**Table 1:** Performance metrics of the internal and external validation dataset.

Matrics	Internal validation metrics(accuracy:0.74)					External validation metrics(accuracy:0.63)				
Factor category	Precision	Recall	F1 score	Accuracy	Specificity	Precision	Recall	F1 score	Accuracy	Specificity
Good	0.85	0.8	0.82	0.738	0.75	0.72	0.68	0.7	0.623	0.6
Medium	0.7	0.65	0.67	0.734	0.68	0.66	0.6	0.63	0.578	0.59
Bad	0.75	0.78	0.76	0.757	0.72	0.7	0.72	0.71	0.619	0.55

Labels “Good” (complete response), “Medium” (partial response), and “Bad” (no response) represent NAC outcomes. Accuracy measures correct classification proportion, precision measures the proportion of true positives among predicted positives, recall measures the proportion of true positives correctly identified, and specificity measures the proportion of true negatives correctly identified.

For non-response prediction, the model also performed robustly, achieving precision, recall, and F1 scores of 0.75, 0.78, and 0.76 in internal validation, with accuracy and specificity of 0.76 and 0.72 (**Table 1**). In external validation, these values slightly decreased to 0.70, 0.72, and 0.71 (**Table 1**). This consistent performance highlights the model’s potential to aid in optimizing treatment strategies for non-responsive patients.

Overall, the model’s performance in predicting both complete and non-response outcomes underscores its clinical applicability, which may be attributed to its strong feature activation and low background noise (see Section 3.2). In contrast, the three comparative models demonstrated inferior performance across all metrics (**Supplementary Data Tables S1–S3**).

#### 3.2.2 Confusion matrix

**Figure 5:**
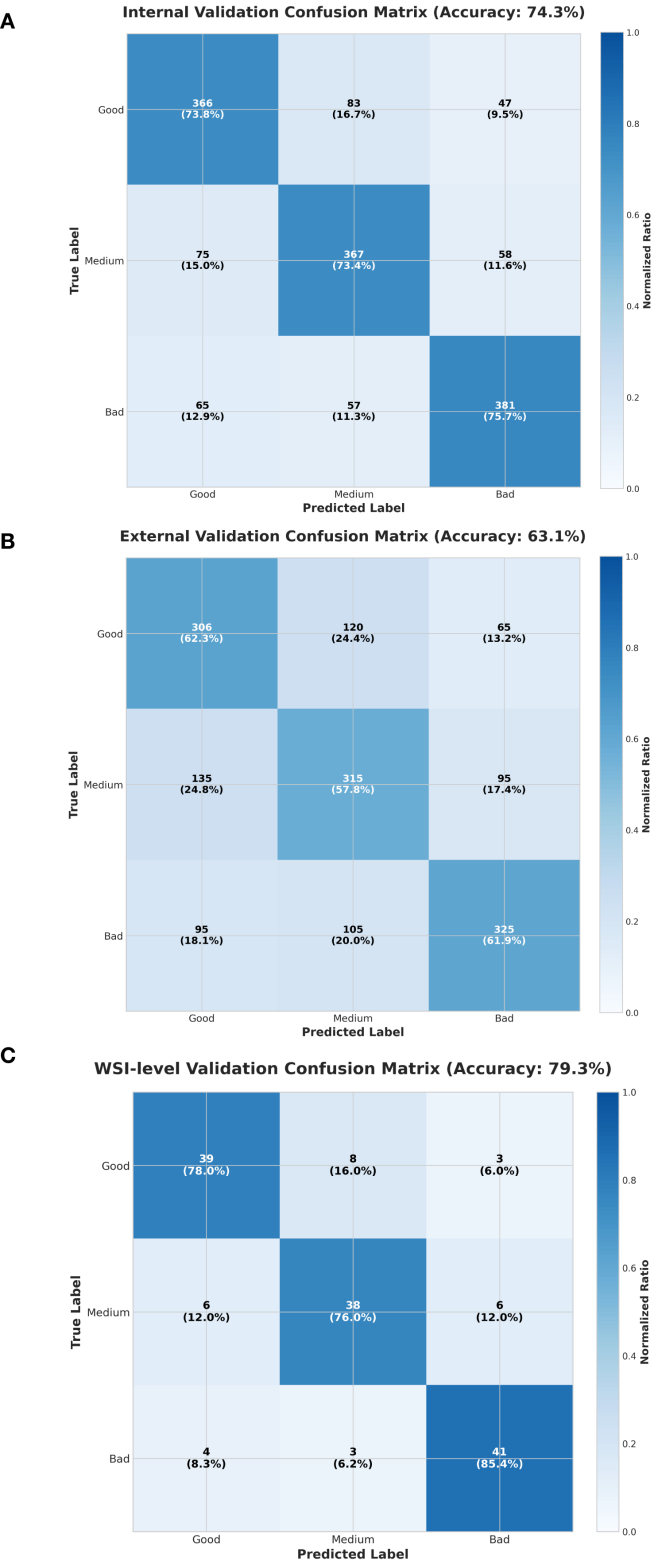
Confusion matrices for three validation settings. **(A)** Internal validation confusion matrix (accuracy: 74.3%). **(B)** External validation confusion matrix (accuracy: 63.1%). **(C)** WSI level validation confusion matrix (accuracy: 79.3%). All matrices are based on three classes—Good, Medium, and Bad—for both true and predicted labels, with normalized ratios indicated by color intensity and percentages shown within each cell.

Confusion matrices were employed to evaluate the model’s classification performance across internal, external, and whole-slide image (WSI)-level validations (**Figure 5**). In the internal validation, the model achieved an overall accuracy of 74.3% (**Figure 5A**), with accuracies of 73.8% and 75.7% for predicting complete response (pCR) and non-response, respectively. Despite domain adaptation challenges, the external validation achieved an overall accuracy of 63.1% (**Figure 5B**), with pCR, non-response, and partial response accuracies of 62.3%, 63.3%, and 57.8%, respectively-representing improvements over the random baseline (33.3%) by 29.0%, 30.0%, and 24.5%. At the WSI level, overall accuracy reached 79.3% (**Figure 5C**), with pCR and non-response accuracies of 78.0% and 85.4%, respectively. These multi-center results highlight the robustness and generalizability of our model.

When compared with alternative architectures, our model achieved the best performance at the WSI level. While ResNet34 demonstrated comparable overall and pCR accuracies (79.3% and 78.0%), it performed slightly worse in predicting non-response (82.0% vs. 85.4%) (**Supplementary Figures S4-2**). All models exhibited decreased performance in external validation, primarily due to the inclusion of 22 microscope images, which introduced additional domain variability and posed greater challenges for generalization from WSIs to standard photographs (**Supplementary Data Figures S4-1**–**S4-3**).

#### 3.2.3 Precision-recall and receiver operating characteristic curves

**Figure 6:**
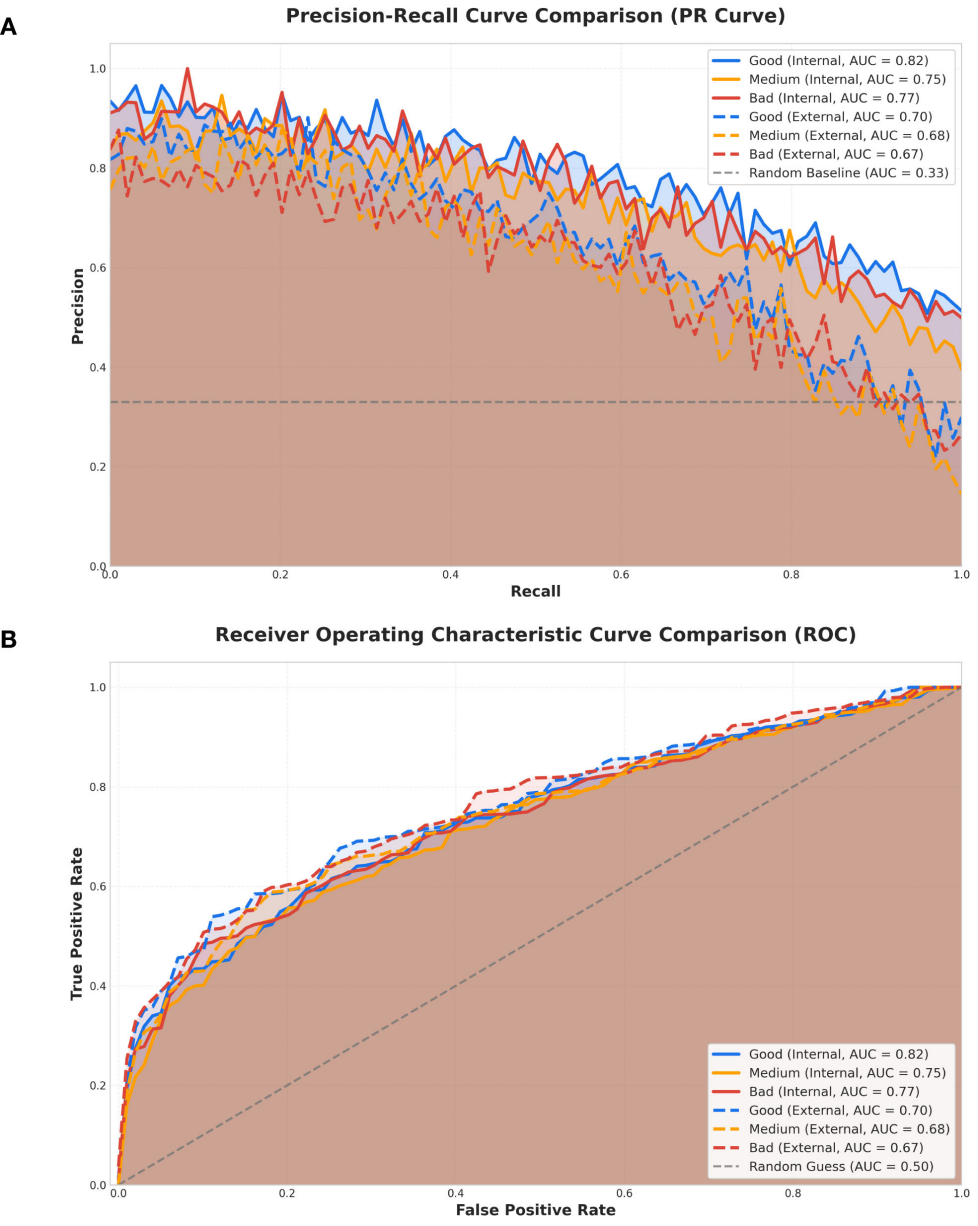
Performance curve comparisons for internal and external validation across three classes. **(A)** Precision–Recall (PR) curves Comparison. PR curves for Good, Medium, and Bad classes under internal and external validation. Area under the curve (AUC) values are indicated in the legend. The dashed horizontal line represents the baseline precision for random classification (AUC = 0.33). **(B)** Receiver Operating Characteristic (ROC) curves Comparison. ROC curves for the same validation settings and classes, with corresponding AUC values shown. The diagonal dashed line indicates the random guess baseline (AUC = 0.50).

Precision-Recall (PR) and Receiver Operating Characteristic (ROC) curves were employed to evaluate the model’s predictive performance (**Figure 6**). PR curves emphasize the precision-recall tradeoff, making them particularly suitable for imbalanced datasets. In internal validation, the PR AUC for complete response (pCR) reached 0.82, exceeding those for partial response (0.75) and non-response (0.77), thereby indicating strong pCR classification capability. In external validation, the corresponding AUCs declined to 0.70, 0.68, and 0.67, suggesting reduced generalization across domains. The random baseline AUC (0.33) was substantially lower, confirming the model’s superior predictive performance. Among the comparison models, the best-performing ResNet34 achieved an internal validation AUC of 0.80 for pCR, which remained slightly inferior to the proposed model (**Supplementary Data Figures S5-2**).

ROC curves, which evaluate overall discrimination across classification thresholds, showed consistent patterns. In internal validation, the AUCs for pCR, partial response, and non-response were 0.82, 0.75, and 0.77, respectively, while in external validation they decreased to 0.70, 0.68, and 0.67. Other models exhibited similar declines in external validation, consistent with the trend observed in our model (**Supplementary Data Figures S5-1**–**S5-3**).

#### 3.2.4 Prediction probability distribution comparison

**Figure 7:**
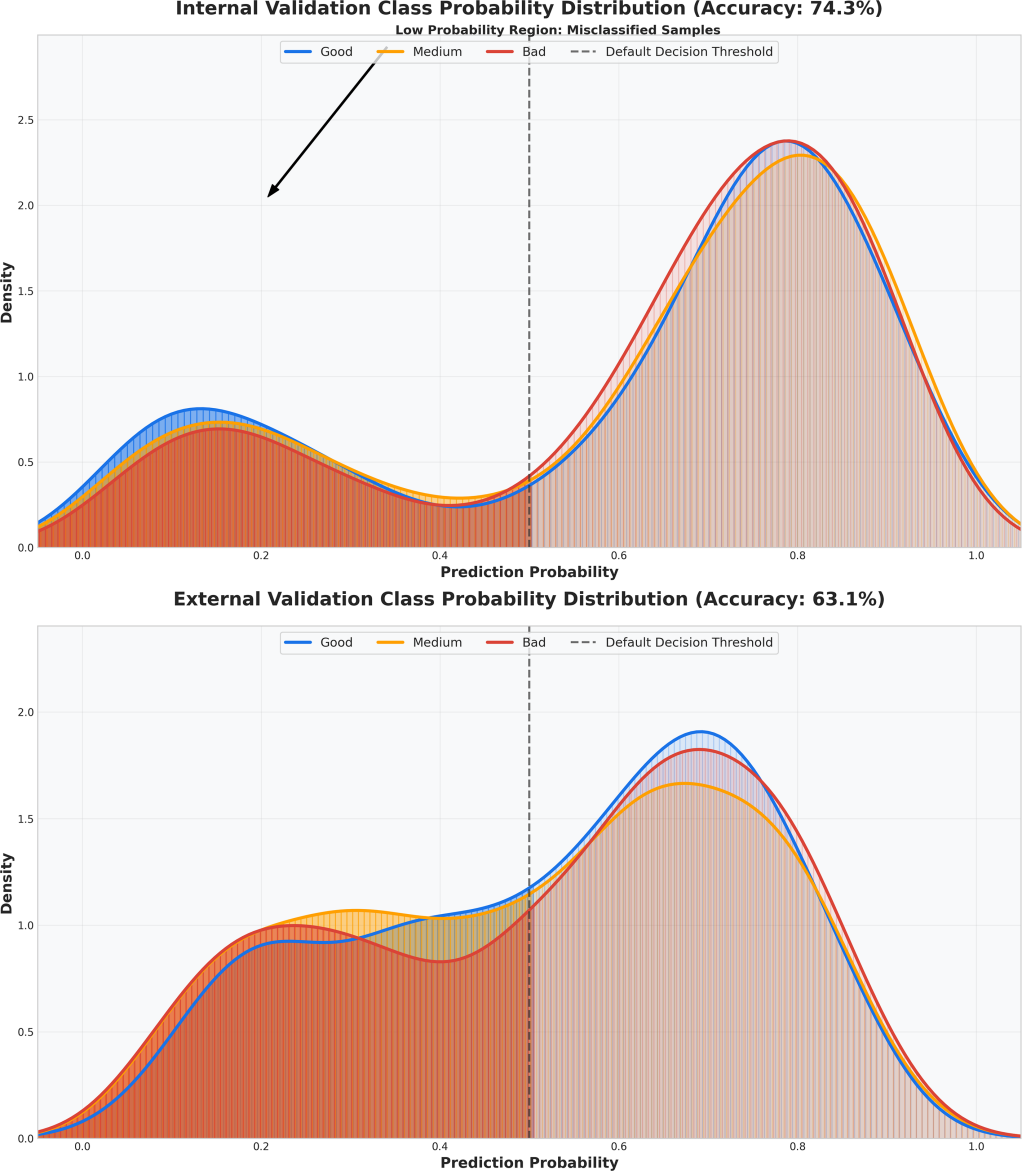
Classification probability distribution plot. The upper half shows probability distributions for complete response, partial response, and non-response in internal validation; the lower half shows external validation, with vertical dashed lines marking default decision thresholds.

Prediction probability distribution plots illustrate the overlap of prediction probabilities in internal and external validation datasets, facilitating decision threshold optimization (**Figure 7**). In internal validation, the model’s accuracy rapidly increased and stabilized around 0.80, while in external validation, it followed a similar trend, reaching a plateau near 0.70-consistently outperforming other models. Probability curves for complete response, partial response, and non-response were closely aligned in internal validation but exhibited slight separation in external validation, reflecting minor domain-induced variability.

### 3.3 Pathological feature activation heatmaps

**Figure 8:**
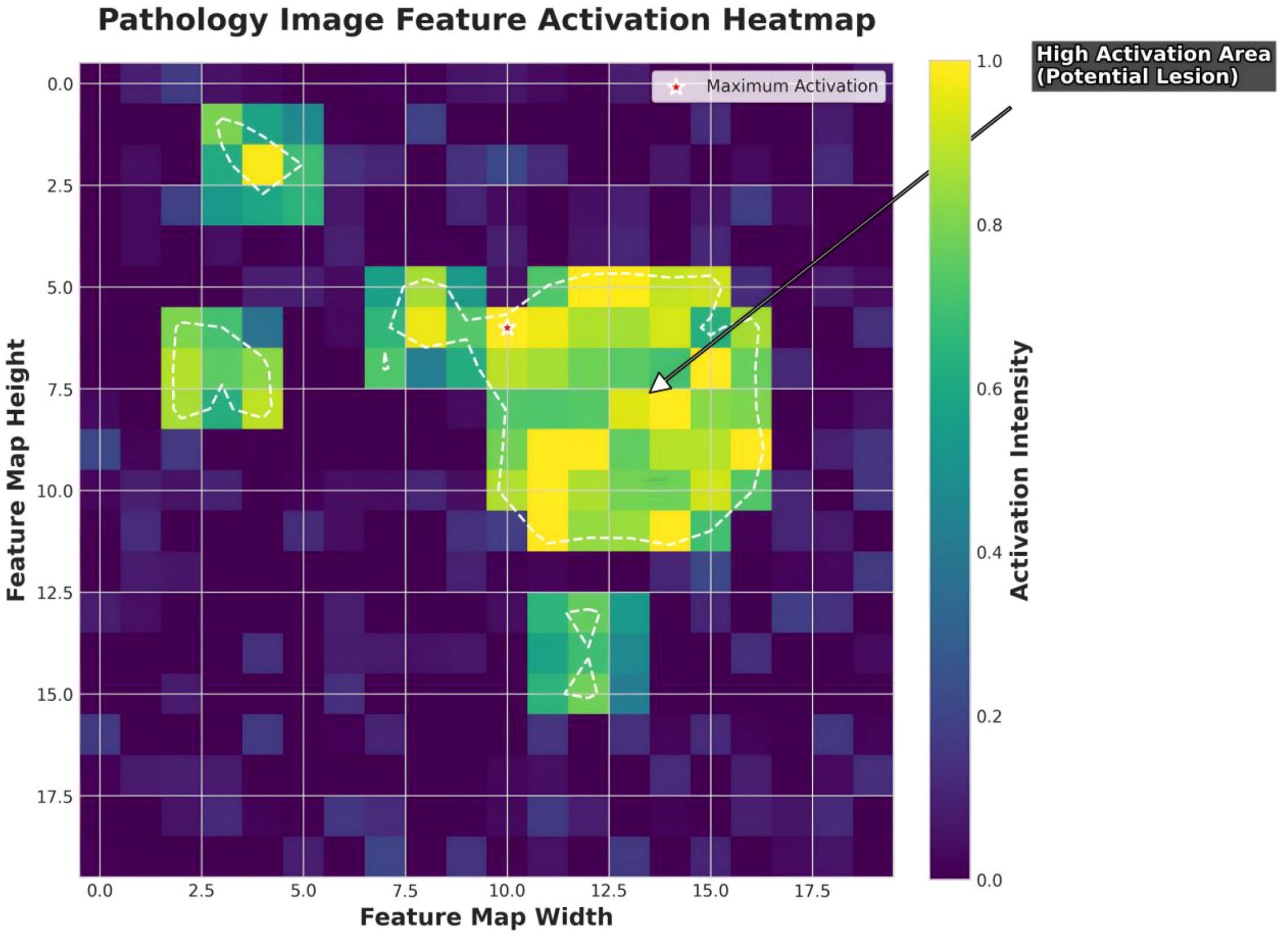
Pathological feature activation heatmap. High-activation regions (ranging from yellow-green to bright green) represent areas of strong model attention, typically corresponding to invasive cancer components. The central circular high-activation zone reflects the primary lesion, while scattered activation points denote secondary but clinically relevant features such as small tumor cell clusters, nuclear atypia, or localized necrosis. White dashed contours delineate activation areas exceeding a defined threshold, marking regions of potential pathological concern. Red star markers indicate maximum activation points, which generally correspond to the most diagnostically significant regions.

Pathological feature activation heatmaps were used to visualize key cancer-detection features learned by the proposed model, facilitating expert interpretation and integration into clinical workflows. On whole-slide images (WSIs), the model exhibited highly focused activation regions with well-defined boundaries and continuous white-dashed contours, precisely delineating potential lesion areas (**Figure 8**). In contrast, GoogleNet produced more rounded but less distinct activations (**Supplemental Data Figure S6-1**); ResNet34 showed good focus but with slightly blurred boundaries (**Supplemental Data Figure S6-2**); and SqueezeNet generated scattered, discrete activation points, indicating poor spatial attention (**Supplemental Data Figure S6-3**).

In terms of signal-to-noise ratio, our model demonstrated minimal background interference and sharp contrast between high-activation regions and the surrounding tissue, facilitating accurate lesion localization (**Figure 8**). GoogleNet showed higher background noise with irrelevant activations (**Supplemental Data Figure S6-1**), ResNet34 displayed moderate noise with partial interference from secondary regions (**Supplemental Data Figure S6-2**), and SqueezeNet exhibited the highest noise level, with numerous random activation points that compromised diagnostic reliability (**Supplemental Data Figure S6-3**).

Regarding activation patterns, our model revealed a coherent central activation zone accompanied by regularly distributed peripheral satellite lesions, consistent with known pathological morphology (**Figure 8**). By comparison, ResNet34 displayed more irregular satellite distributions (**Supplemental Data Figure S6-2**), GoogleNet focused on a single dominant region and overlooked minor features (**Supplemental Data Figure S6-1**), and SqueezeNet produced chaotic, clinically inconsistent activations (**Supplemental Data Figure S6-3**). Moreover, our model’s high-activation regions (bright yellow) were extensive and uniform, indicating confident and stable lesion identification (**Figure 8**), whereas ResNet34 exhibited less uniform activation intensity (**Supplemental Data Figure S6-2**), GoogleNet showed moderate intensity with weak edge responses (**Supplemental Data Figure S6-1**), and SqueezeNet presented low and uneven intensities, reducing localization reliability (**Supplemental Data Figure S6-3**).

## 4 Discussion

The proposed model, developed on the TransMIL framework, performed satisfactorily in predicting neoadjuvant chemotherapy (NAC) response in breast cancer. Internal validation achieved an accuracy of 74.3%, WSI-level accuracy reached 79.3%, and external validation attained 63.1%, outperforming GoogleNet, ResNet34, and SqueezeNet, further demonstrating the feasibility of the model. The model showed favorable performance in predicting complete response (pCR, Grade 5) with an AUC of 0.82. External validation using lower-resolution microscope images further supports its real-world clinical applicability. The model integrates ResNet50 and Transformer encoders, where multi-scale attention effectively captures both local (nuclear atypia) and global (tumor microenvironment) features, surpassing conventional CNNs such as ResNet34 and GoogleNet that primarily emphasize local patterns. Heatmap analyses (**Figure 6**) revealed distinct lesion boundaries and high signal-to-noise ratios, further demonstrate clinical interpretability. A novel two-stage classification strategy-first differentiating “Medium” from “non-Medium”, then separating “Good” from “Bad”-reduces class confusion and enhances focus on chemotherapy-resistant patients prone to toxicity, a topic seldom addressed in previous research.

Clinically, accurate prediction of NAC response holds substantial importance. Recent studies have reported higher quality-of-life scores in non-surgical patients, with 90% satisfaction and lower anxiety or depression rates at three-year follow-up (3, 19, 20). Local recurrence remains below 5%, and no distant metastases have been reported (19, 20). Although clinicians increasingly consider omitting surgery after pCR, imaging-guided biopsy false-negative rates (5-15%) (21) limit adoption, and ongoing trials progress slowly (22). Existing trials (19, 20, 23–25) involve small cohorts and short follow-up periods (<3 years), leaving long-term safety uncertain. Therefore, accurate pCR assessment is essential. Our model’s strong predictive performance (AUC 0.82 internally; 0.70 externally) underscores its reliability in identifying chemotherapy-insensitive subgroups. This could guide alternative treatments for HR+/HER2- low-proliferation breast cancers (26), early surgical candidates (27), patients at high risk of toxicity, or those with low chemotherapy benefit based on genomic assays (28). The model reduces inter-observer variability, enhances diagnostic consistency, and supports personalized treatment planning.

Recently, some studies have continued to expand the scope of computational pathology in NAC prediction. Large-scale multicenter investigations such as Mao et al. (17) and Zhou et al. (18) have introduced multimodal and interpretable AI systems, achieving improved robustness across centers and imaging modalities. Compared with these recent multimodal approaches, our model’s external validation (AUC 0.70) highlights the remaining challenges of cross-domain generalization and domain shift in pathological imaging. Image quality variations such as defocus blur and inconsistent focal planes are known to affect WSI-based classification performance (29), which may partially explain the performance degradation observed in our external microscope-image validation. The limited size of the external cohort and potential overfitting to WSI-specific features may also contribute to this issue.

From a methodological standpoint, the proposed framework integrates multiple advanced components-specifically the two-stage classifier, Domain Adversarial Neural Networks (DANN) and metric learning-to address the specific challenges of pathological image analysis. While we acknowledge that a quantitative ablation study was not performed to isolate the numerical contribution of each module, the inclusion of these components is grounded in the distinct characteristics of our clinical dataset. First, the two-stage classification strategy was essential to address the inherent class ambiguity between ‘Partial’ and ‘No’ response groups. Given the severe class imbalance (only 17 ‘Poor’ responders), a standard single-stage classifier would likely bias towards the majority class; the two-stage approach effectively mimics clinical triage, prioritizing the identification of distinct response patterns. Second, the integration of DANN was necessitated by the significant domain shift between the high-resolution digital WSIs used for training and the lower-resolution microscope images used for external validation. Without this domain adaptation mechanism, the model’s generalization to resource-limited settings-a key objective of this study-would be theoretically compromised. Finally, metric learning was employed to enforce feature separability in the latent space, which is critical when training on small-sample datasets to prevent overfitting. We posit that given the limited sample size (N = 128), treating the system as a holistic solution ensures stability, whereas removing individual components for ablation might introduce stochastic noise. Future investigations with larger, multi-center cohorts will allow for a more granular decomposition of these architectural contributions.

This study still has several limitations. Firstly, Although merging Miller-Payne grades 2–4 into a unified “Medium” category facilitates model training and reduces class imbalance, this simplification may obscure biologically meaningful heterogeneity within the intermediate response spectrum. Grades 2–4 represent a continuum of tumor chemosensitivity, encompassing minimal, moderate, and near-complete reductions in tumor cellularity. These differences have been associated with distinct patterns of immune activation, stromal remodeling, proliferative activity, and molecular pathways related to DNA damage repair and drug metabolism. Consequently, collapsing these grades into a single category may dilute subtle yet clinically relevant biological signals. Moreover, the Medium group itself is clinically heterogeneous. Misclassification of this category may lead to suboptimal treatment decisions: overestimating response (Medium→Good) may delay necessary regimen escalation or influence surgical planning inappropriately, whereas underestimating response (Medium→Bad) may prompt unnecessary treatment intensification and increased toxicity.

Secondly, the dataset is limited in size and exhibits an imbalanced class distribution, with the ‘Bad’ category being particularly underrepresented. While the application of SMOTE and ADASYN was instrumental in counteracting the severe class imbalance inherent to our clinical cohort, we explicitly acknowledge the methodological nuances and potential limitations associated with synthetic oversampling in the high-dimensional feature space. A primary concern is that these algorithms rely on linear interpolation between nearest neighbors, a process that may not fully preserve the complex, non-linear manifold structure of histopathological embeddings or the intricate inter-patch dependencies unique to the MIL paradigm. Theoretically, this could introduce artificial correlations or synthetic feature vectors that deviate from biologically plausible representations. However, to rigorously guard against the risk of optimistic bias, we restricted these augmentation strategies exclusively to the training partitions of our cross-validation framework. By ensuring that no synthetic data leaked into the validation or independent testing sets, we guarantee that the reported performance metrics are derived solely from authentic, unseen patient data. This strict separation validates that the observed performance gains reflect genuine improvements in the model’s ability to generalize to minority class examples, rather than artifacts arising from synthetic oversampling. Future work should expand sample size and explore advanced rebalancing methods (e.g., focal loss (30)) to improve minority class recognition.

Moreover, although ResNet50 performs effectively for patch-level feature extraction, it may not fully leverage the capabilities of recently developed pathology foundation models such as Virchow2 (31, 32), GigaPath (33), and H-Optimus-1 (34), which have demonstrated superior feature representation in large-scale histopathological benchmarks. Similarly, emerging WSI-specific MIL frameworks, including CLAM (35), DSMIL (36), and HIPT (37), have become important benchmark methods in this field. As the dataset expands in future studies, we will incorporate these models into systematic comparisons to achieve more comprehensive and convincing performance evaluations.

Despite these limitations, the present study provides a practical and interpretable baseline for NAC response prediction from pre-treatment biopsy slides. Its simplicity and reliance on a single imaging modality make it potential deployable for real-world deployment in clinical settings with limited resources. Moreover, ensemble learning and multi-scale attention mechanisms ensure prediction basic stability and a certain degree of interpretability, while the two-stage strategy provides clinically meaningful stratification that may guide individualized treatment decisions. By integrating larger multi-institutional datasets and advanced feature extractors, future iterations of this model could bridge the gap between research performance and clinical translation.

## 5 Conclusion

This study proposes an integrated Transformer-based multiple instance learning model for predicting the efficacy of neoadjuvant chemotherapy in breast cancer patients. By incorporating a multi-scale attention mechanism, a two-stage classification strategy, and cross-modal adaptation techniques, the model outperforms traditional convolutional neural networks across multiple evaluation metrics, demonstrating discriminative ability in identifying pathological complete response (pCR) and non-responsive patients. Relying solely on pre-treatment whole slide images, the method offers a measurable level of applicability and generalizability, particularly for resource-limited clinical settings. External validation results further confirm the model’s potential clinical utility, although slight performance degradation was observed on microscope images. Future improvements can be achieved by expanding data scale and incorporating multimodal information to enhance the model’s generalization, providing more precise and intelligent support for personalized breast cancer treatment.

## Data Availability

The raw data supporting the conclusions of this article will be made available by the authors, without undue reservation.
