# Supplementary material for: Ensemble transformer-based multiple instance learning for predicting neoadjuvant chemotherapy response from breast cancer biopsy whole-slide images
Source: Front Oncol. 2026 Feb 23;16:1728511. doi: 10.3389/fonc.2026.1728511 (PMC12967991; doi:10.3389/fonc.2026.1728511)
Supplement: Supplementary file 1 [file DataSheet1.docx]

**SUPPLEMENTAL DATA**

**Multi-instance learning based on integrated Transformer for predicting neoadjuvant therapy efficacy from whole-slide images of breast cancer biopsy pathology**

Zhenshui Wu^1†^ , Kaining Ye^2†^, Jianming Weng^2^, Zhongping Zhang^2^, Xuehong Liao^3*^ and Kaixin Du^4*^

1. Department of Pathology, The Second Affiliated Hospital of Fujian Medical University, Quanzhou, Fujian, China.
2. Department of Radiation Oncology, Fujian Medical University Xiamen Humanity Hospital, Xiamen, China.
3. Department of Pathology, Zhangzhou Affiliated Hospital of Fujian Medical University, Zhangzhou,Fujian, China.
4. Department of Pathology, Sapporo Medical University, Sapporo, Japan.

**Contents**

Figure S1-1 GoogleNet Validation Accuracy and Area Under the Curve(AUC) Comparison.

Figure S1-2 GoogleNet Class-Specific Training Loss Curves

Figure S2-1 ResNet Validation Accuracy and Area Under the Curve(AUC) Comparison.

Figure S2-2 ResNet34 Class-Specific Training Loss Curves

Figure S3-1 SqueezeNet Validation Accuracy and Area Under the Curve(AUC) Comparison.

Figure S3-2 SqueezeNet Class-Specific Training Loss

Figure S4-1 GoogleNet Confusion Matrix Showing Prediction vs. True Label Matches.

Figure S4-2 ResNet34 Confusion Matrix Showing Prediction vs. True Label Matches.

Figure S4-3 SqueezeNet Confusion Matrix Showing Prediction vs. True Label Matches.

Figure S5-1 GoogleNet Precision-Recall (PR) and Receiver Operating Characteristic(ROC) Curves Comparison.

Figure S5-2 ResNet Precision-Recall (PR) and Receiver Operating Characteristic(ROC) Curves Comparison.

Figure S5-3 SqueezeNet Precision-Recall (PR) and Receiver Operating Characteristic(ROC) Curves Comparison.

Figure S6-1 GoogleNet Feature Activation Heatmap for Histopathology Image.

Figure S6-2 ResNet Feature Activation Heatmap for Histopathology Image.

Figure S6-3 SqueezeNet Feature Activation Heatmap for Histopathology Image.

Figure S1-1. GoogleNet Validation Accuracy and Area Under the Curve(AUC) Comparison.


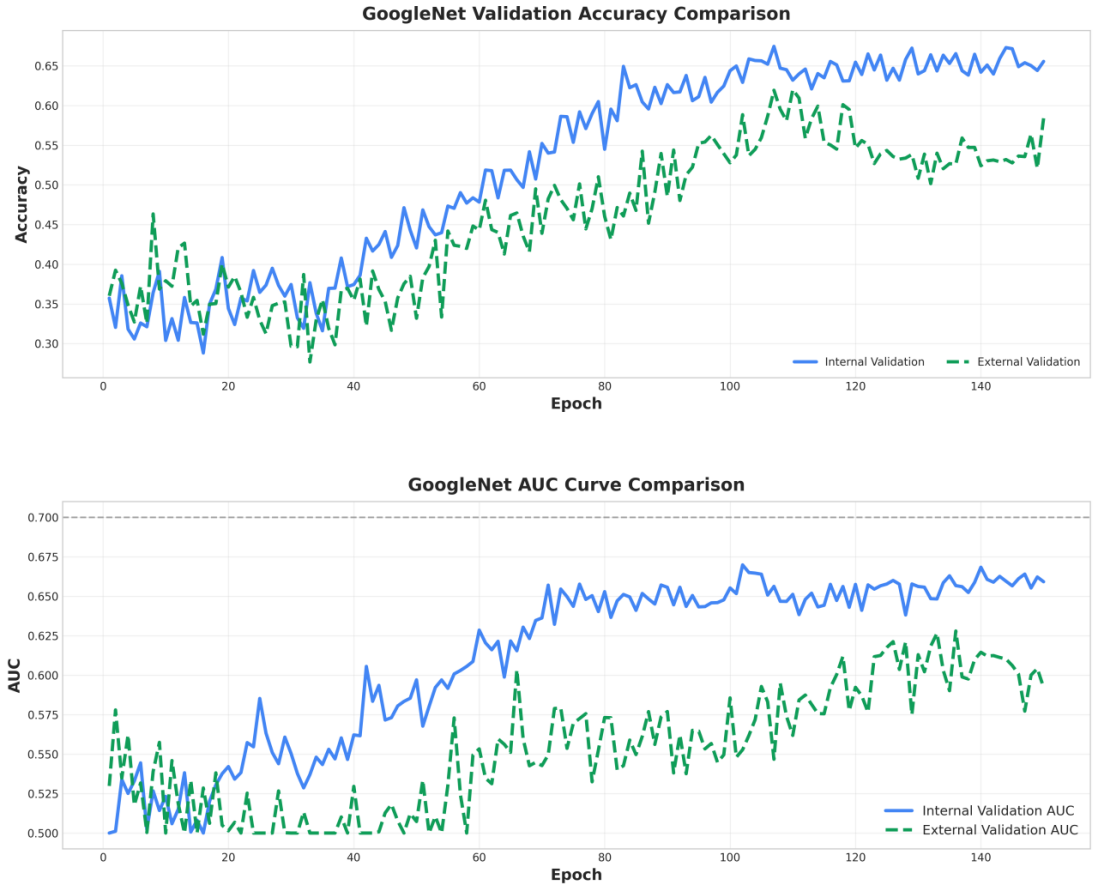


Figure S1-1 This figure compares the validation accuracy and AUC of the GoogleNet model across different training epochs, illustrating its performance stability and discriminative capability.

Figure S1-2. GoogleNet Class-Specific Training Loss Curves


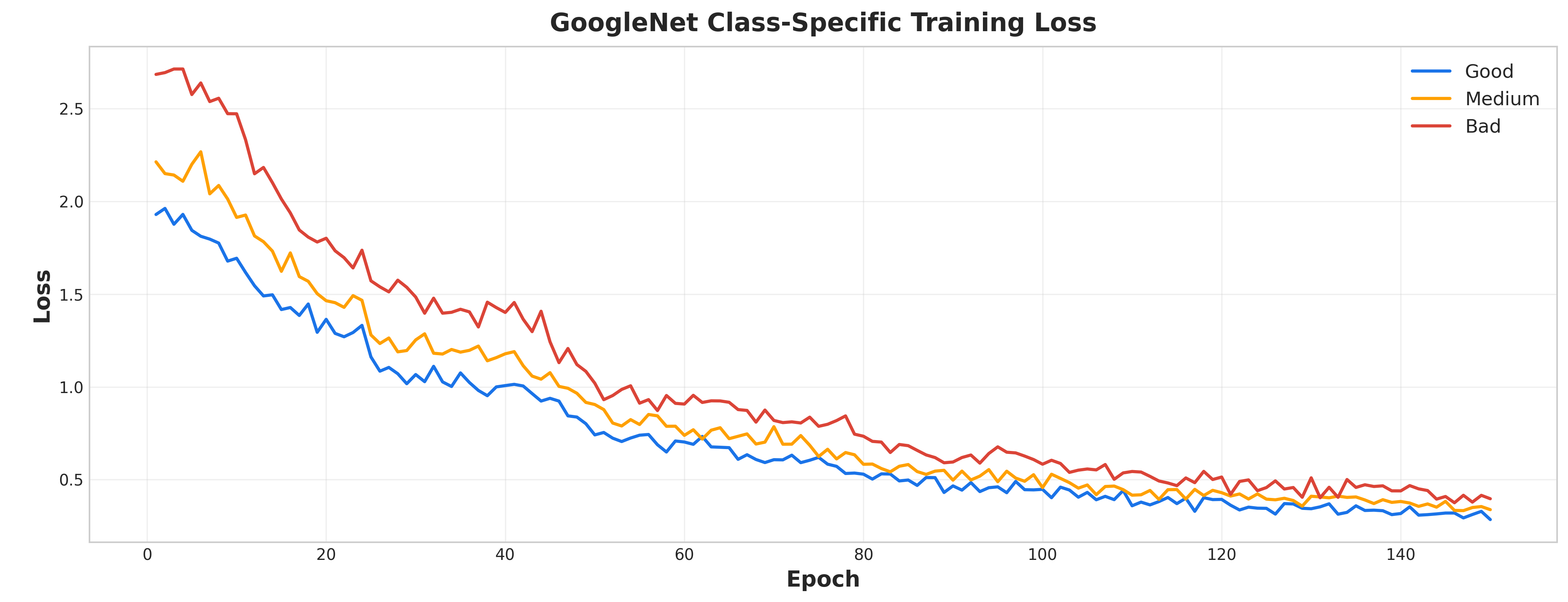


Figure S1-2 Class-wise training loss curves of GoogleNet showing the convergence behavior for each category during model training.

Figure S2-1 ResNet Validation Accuracy and Area Under the Curve(AUC) Comparison.


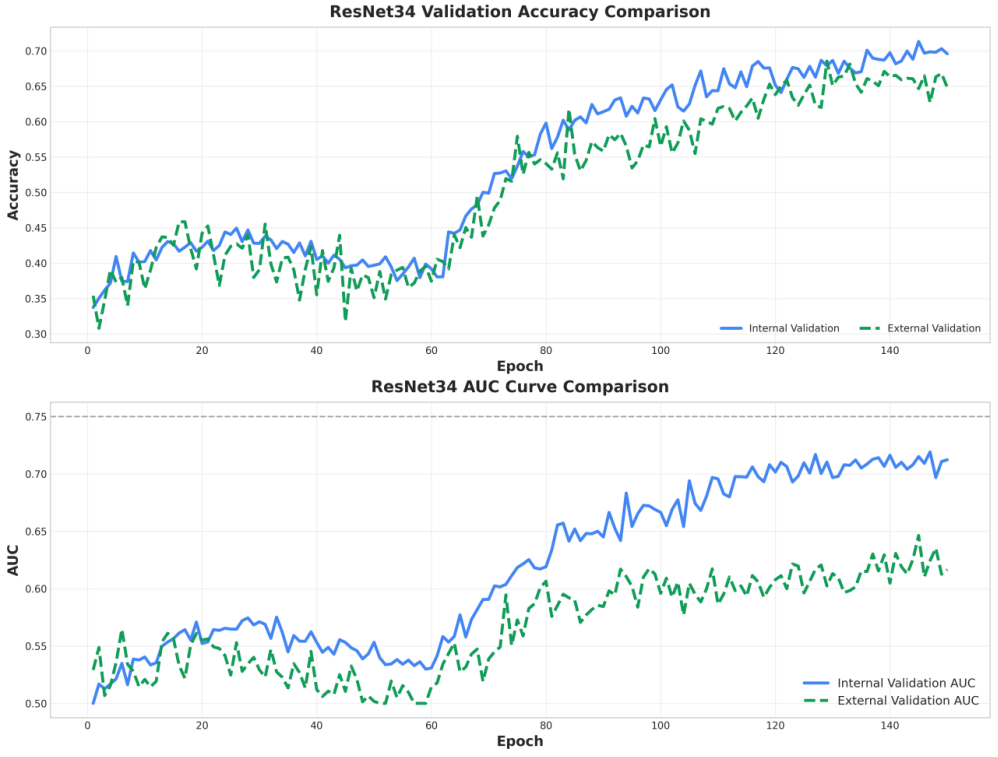


Figure S2-1 Validation accuracy and AUC trends of the ResNet34 model across epochs, demonstrating its predictive performance on the validation dataset.

Figure S2-2 ResNet34 Class-Specific Training Loss Curves


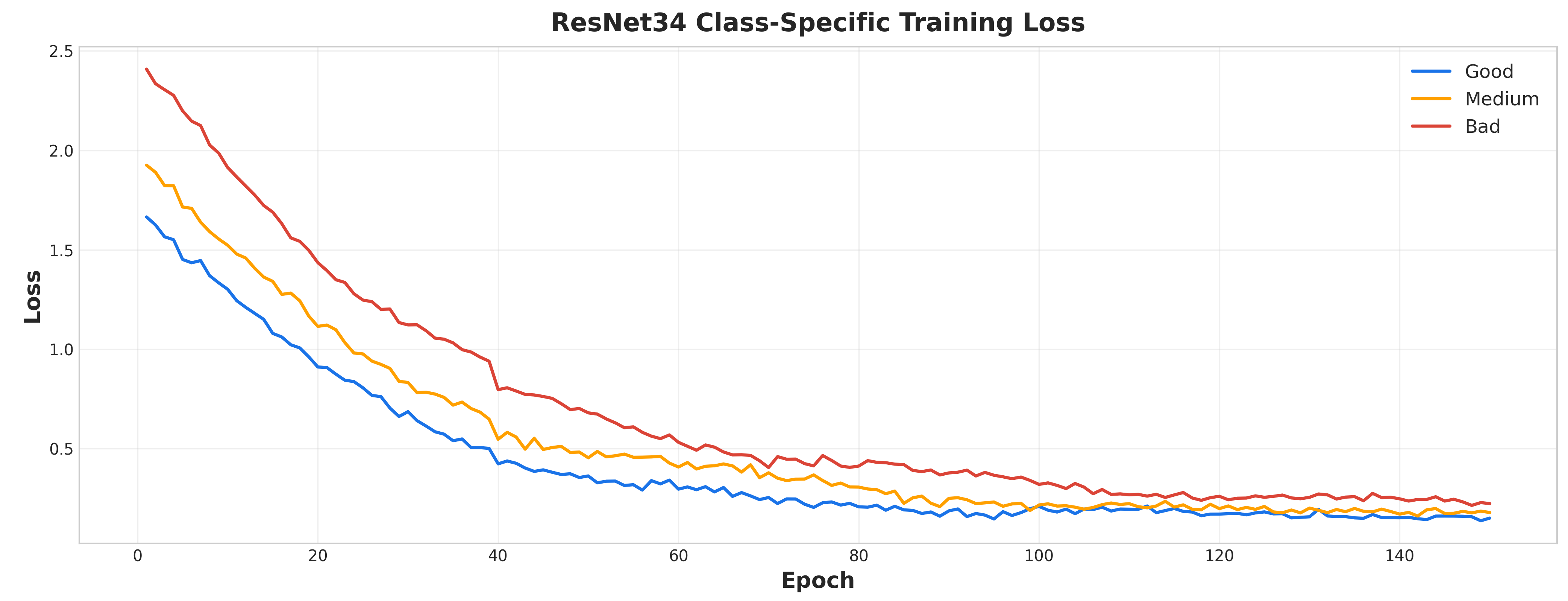


Figure S2-2 Training loss curves for each class using ResNet34, reflecting the model’s learning progression and class-level optimization.

Figure S3-1 SqueezeNet Validation Accuracy and Area Under the Curve(AUC) Comparison.
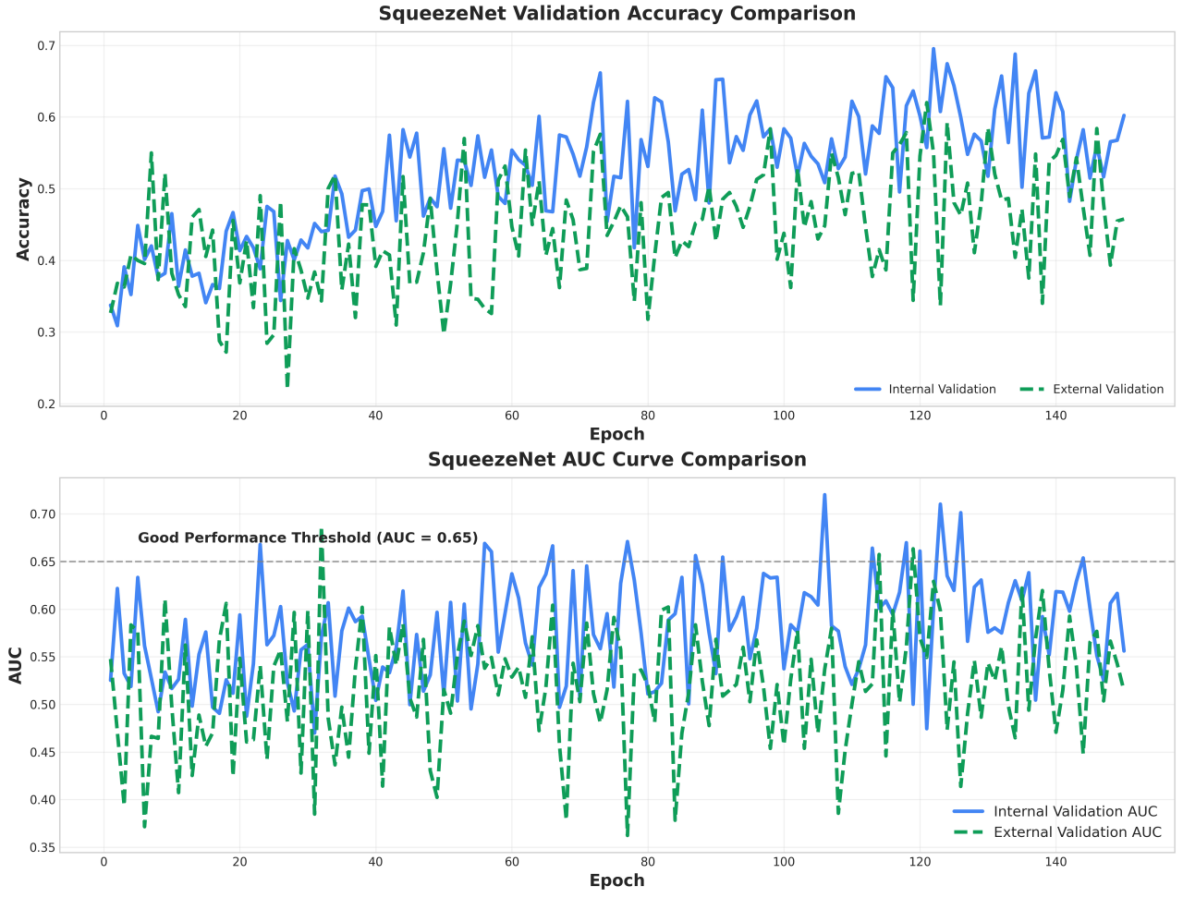


Figure S3-1 Validation accuracy and AUC comparison for SqueezeNet, highlighting its performance characteristics during training.

Figure S3-2 SqueezeNet Class-Specific Training Loss


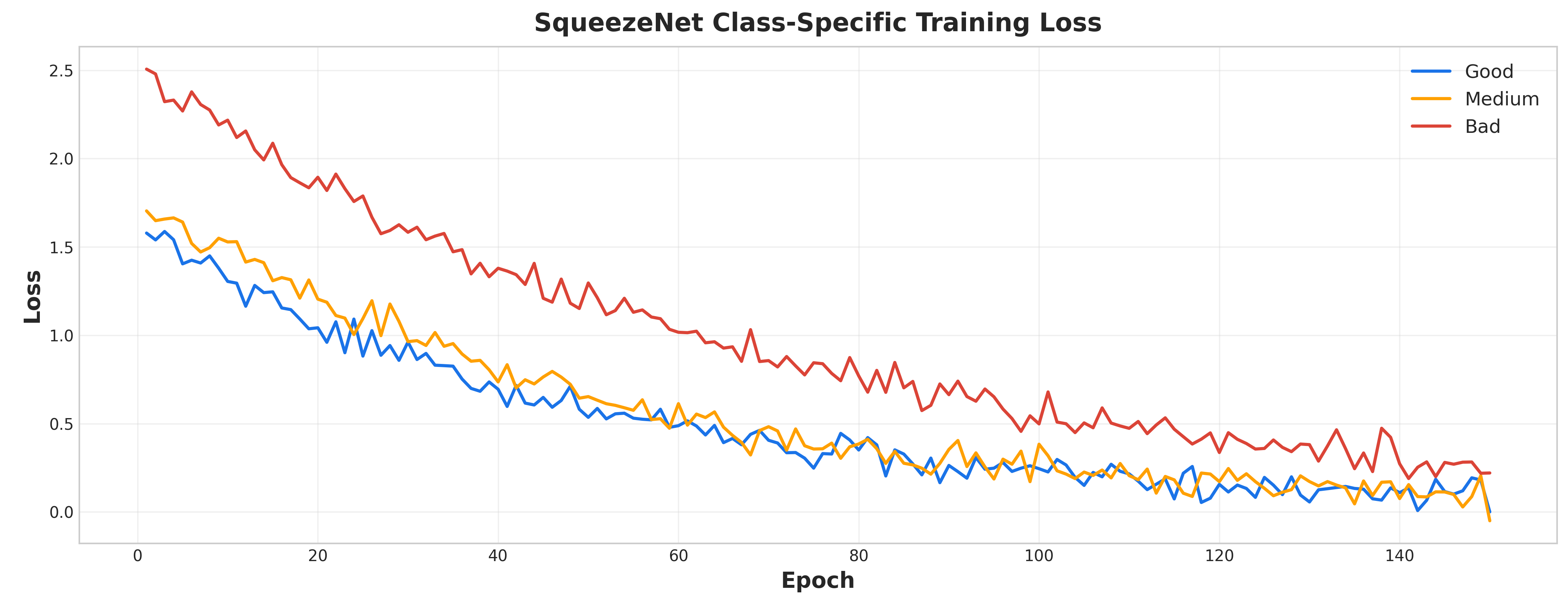


Figure S3-2 Class-specific training loss curves of SqueezeNet, showing the model’s convergence patterns for each category.

Figure S4-1. GoogleNet Confusion Matrix Showing Prediction vs. True Label Matches.


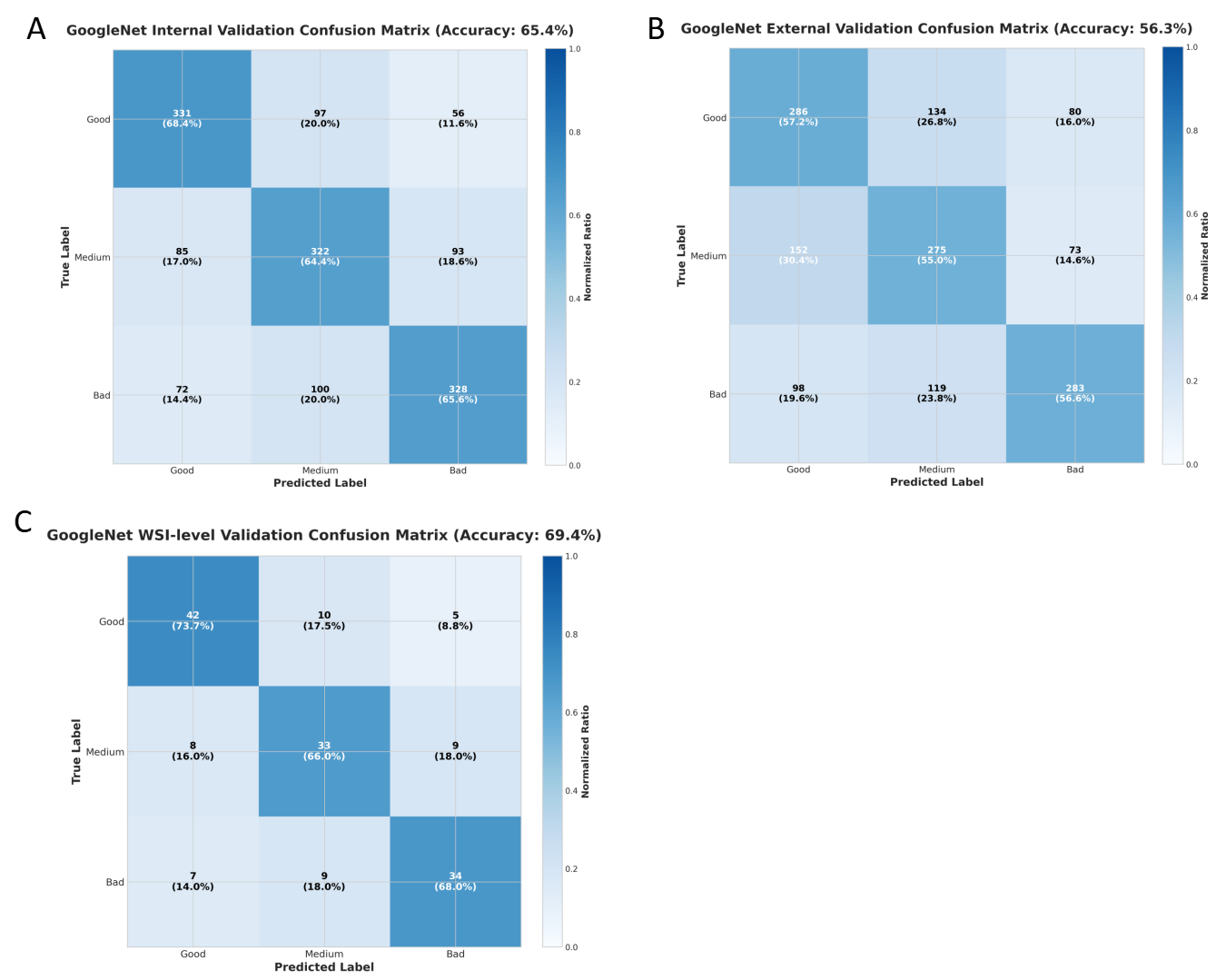


Figure S4-1 Confusion matrix of GoogleNet illustrating the distribution of correct and incorrect predictions across all classes.

Figure S4-2. ResNet34 Confusion Matrix Showing Prediction vs. True Label Matches.


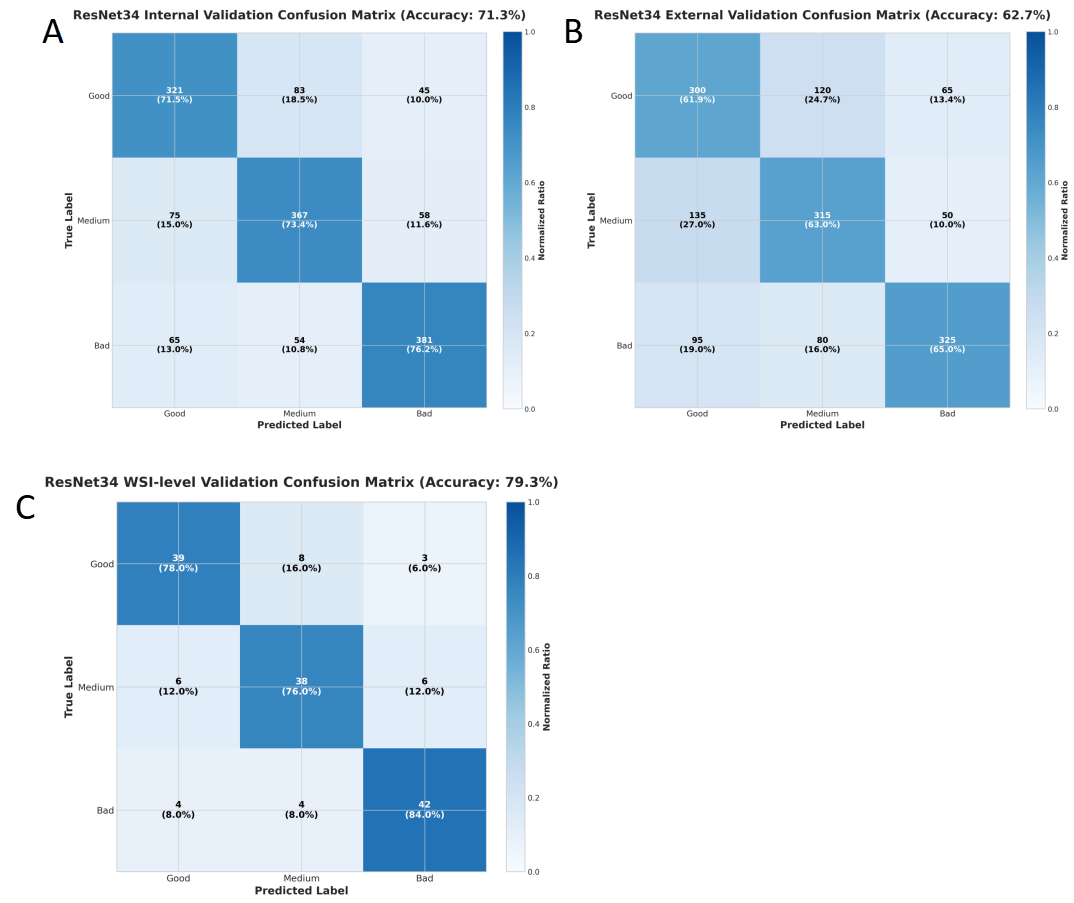


Figure S4-2 Confusion matrix of ResNet34 showing class-wise prediction accuracy and misclassification patterns.

Figure S4-3 SqueezeNet Confusion Matrix Showing Prediction vs. True Label Matches.


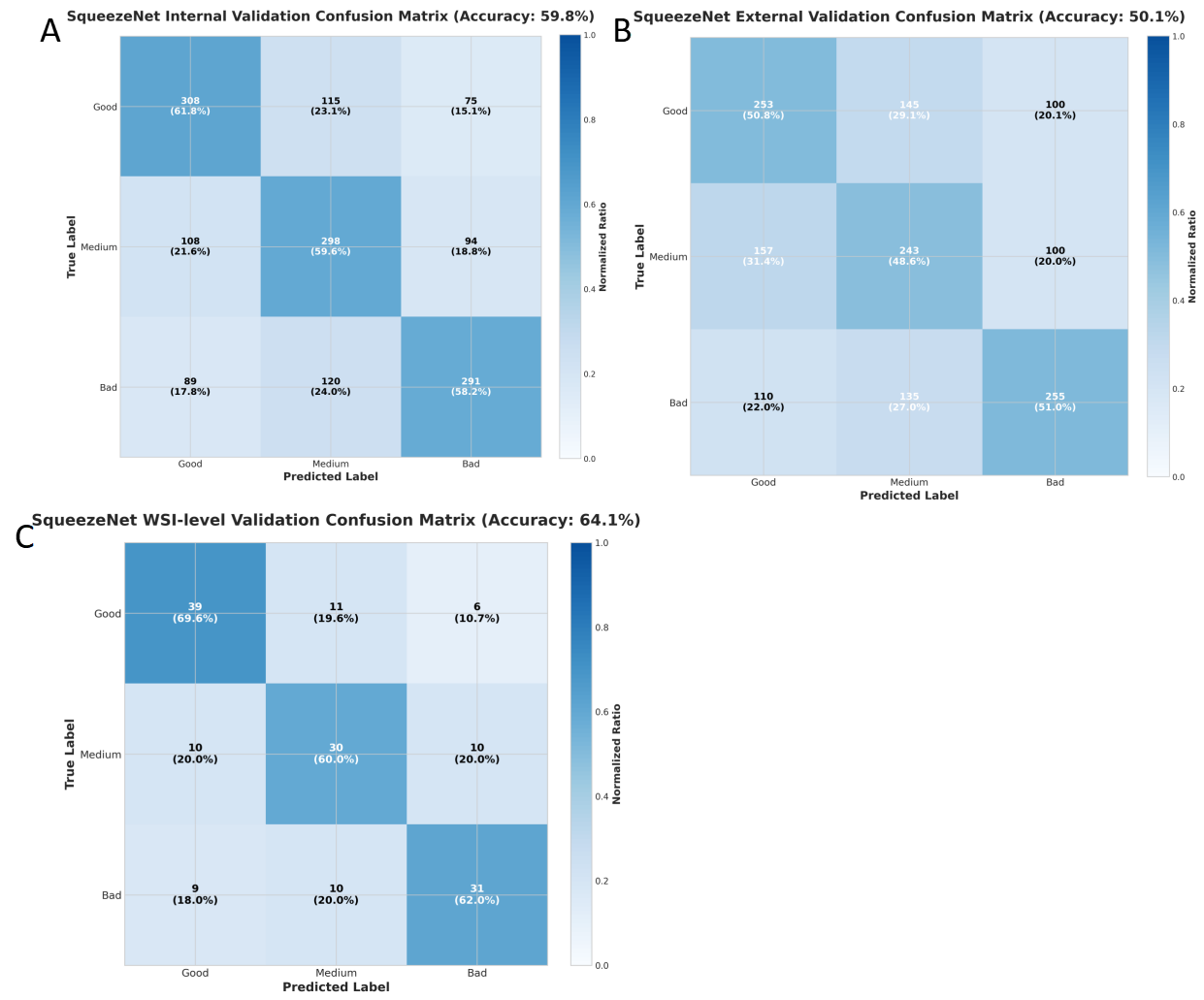


Figure S4-3 Confusion matrix of SqueezeNet depicting the agreement between predicted and true labels for each class.

Figure S5-1. GoogleNet Precision-Recall (PR) and Receiver Operating Characteristic(ROC) Curves Comparison.


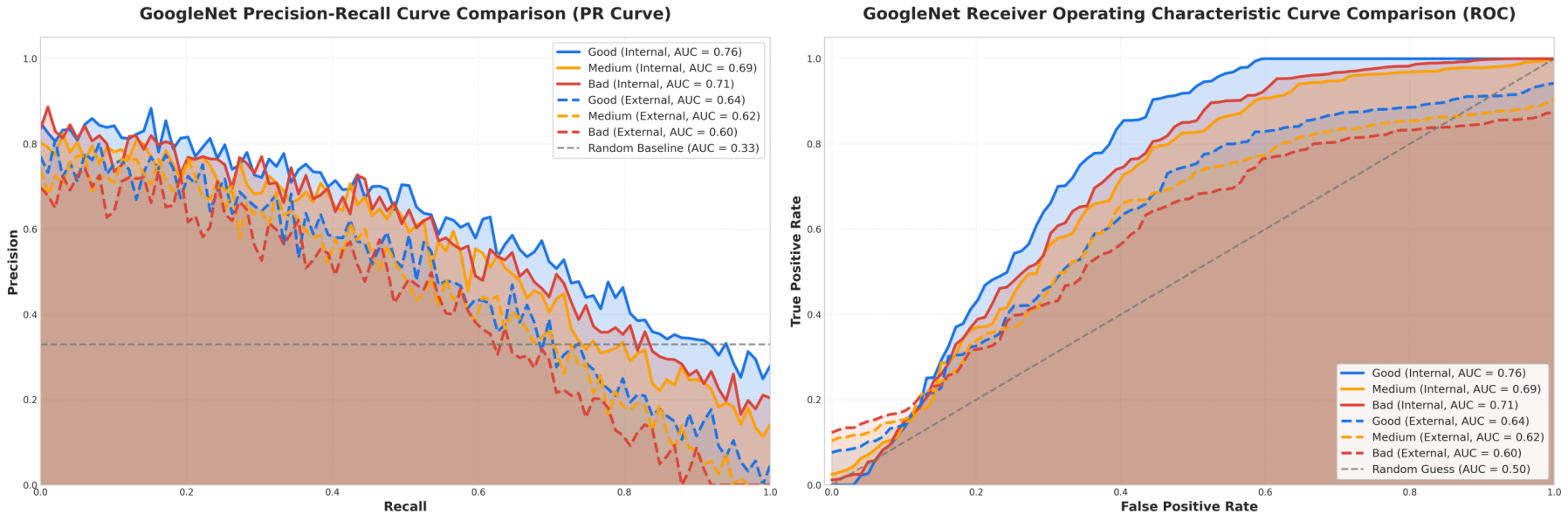


Figure S5-1 PR and ROC curves of GoogleNet demonstrating its sensitivity–specificity balance and precision–recall performance.

Figure S5-2. ResNet Precision-Recall(PR) and Receiver Operating Characteristic(ROC) Curves Comparison.


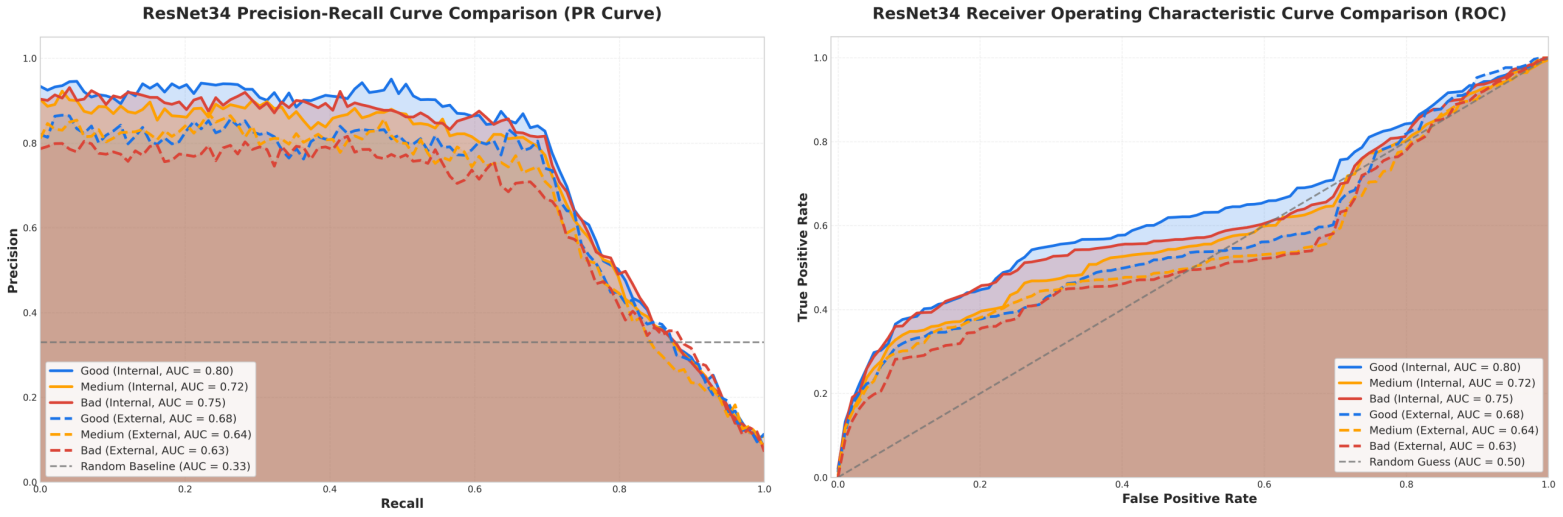


Figure S5-2. PR and ROC curves of ResNet34 showing its classification performance across varying thresholds.

Figure S5-3 SqueezeNet Precision-Recall (PR) and Receiver Operating Characteristic(ROC) Curves Comparison.


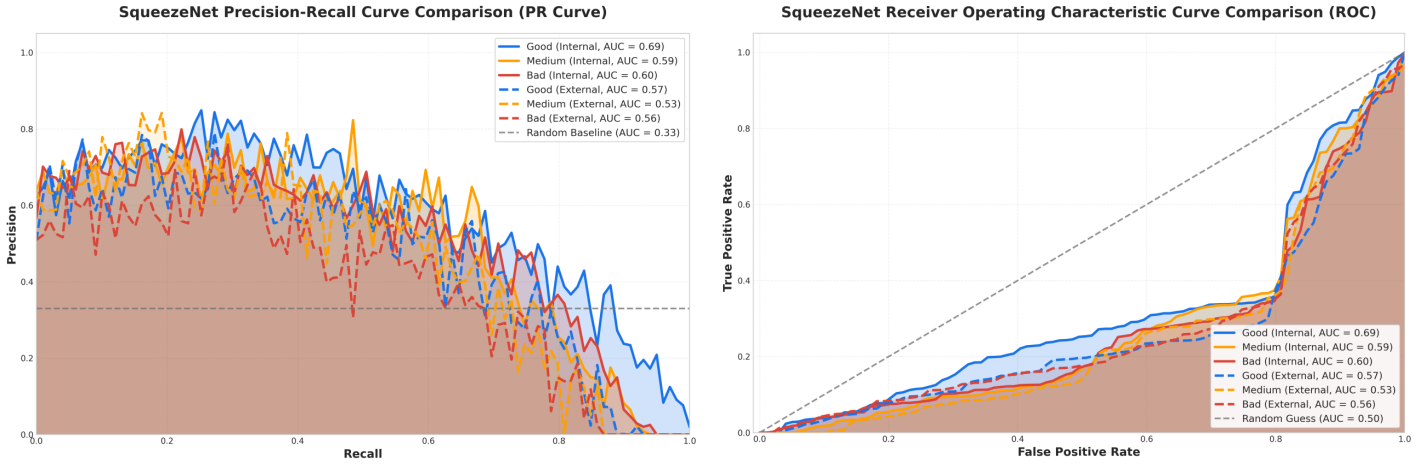


Figure S5-3 PR and ROC curves of SqueezeNet illustrating its threshold-dependent classification behavior.

Figure S6-1 GoogleNet Feature Activation Heatmap for Histopathology Image


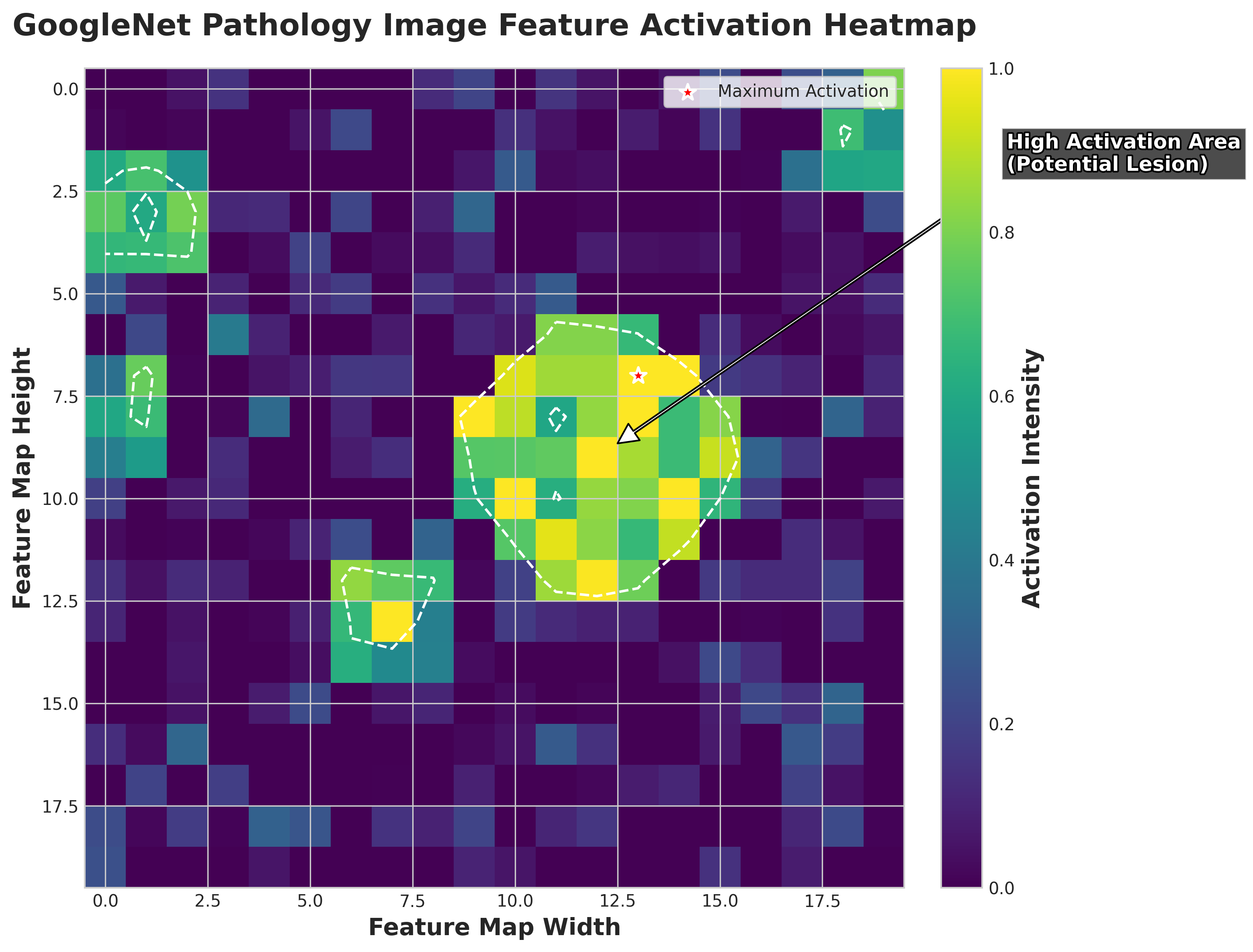


Figure S6-1 Heatmap showing GoogleNet’s activation intensity across the histopathology image, highlighting regions contributing most to the model’s decision.

Figure S6-2 ResNet Feature Activation Heatmap for Histopathology Image


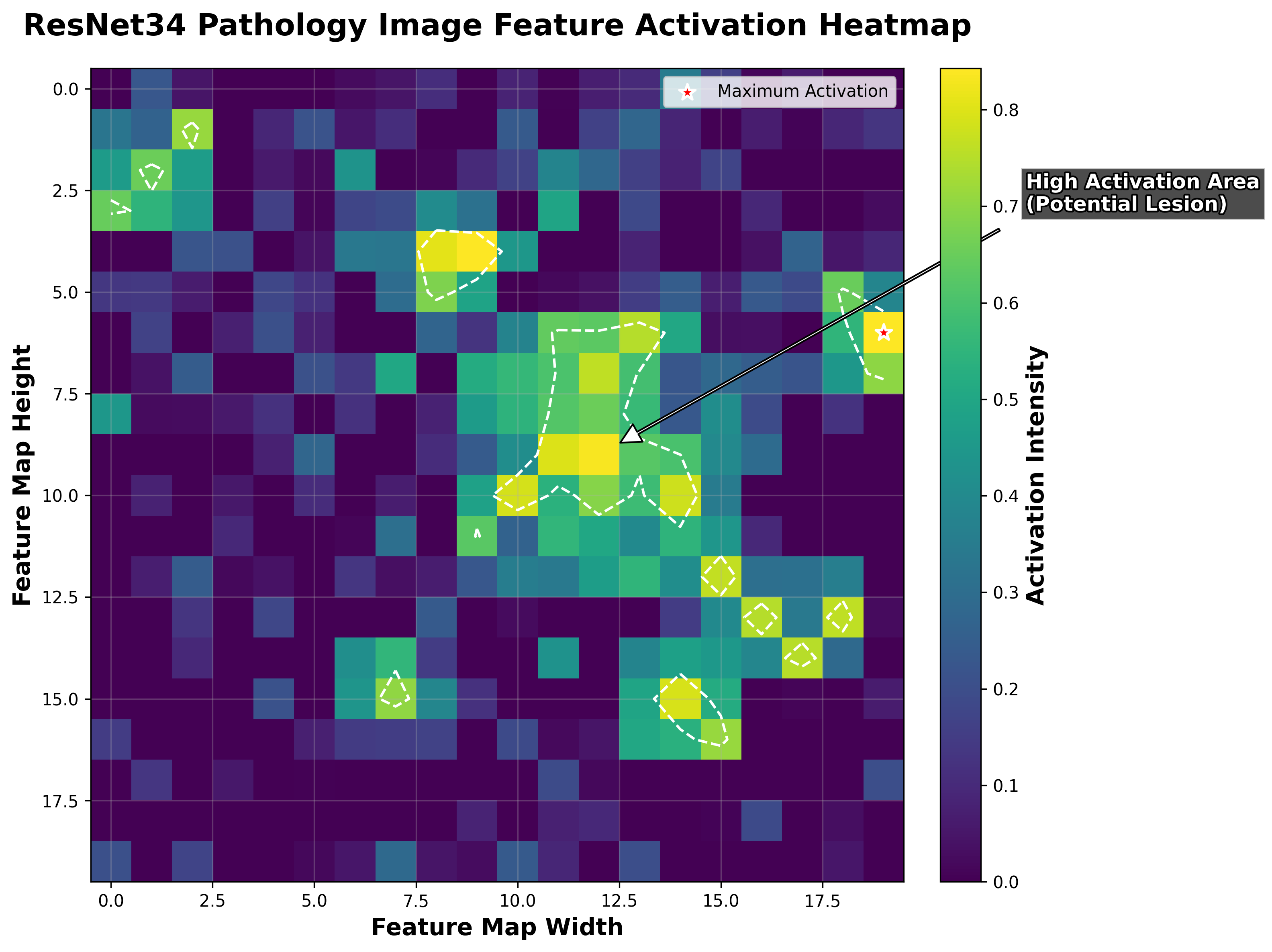


Figure S6-2 Feature activation heatmap of ResNet34 indicating the spatial regions with the highest relevance during inference.

Figure S6-3 SqueezeNet Feature Activation Heatmap for Histopathology Image


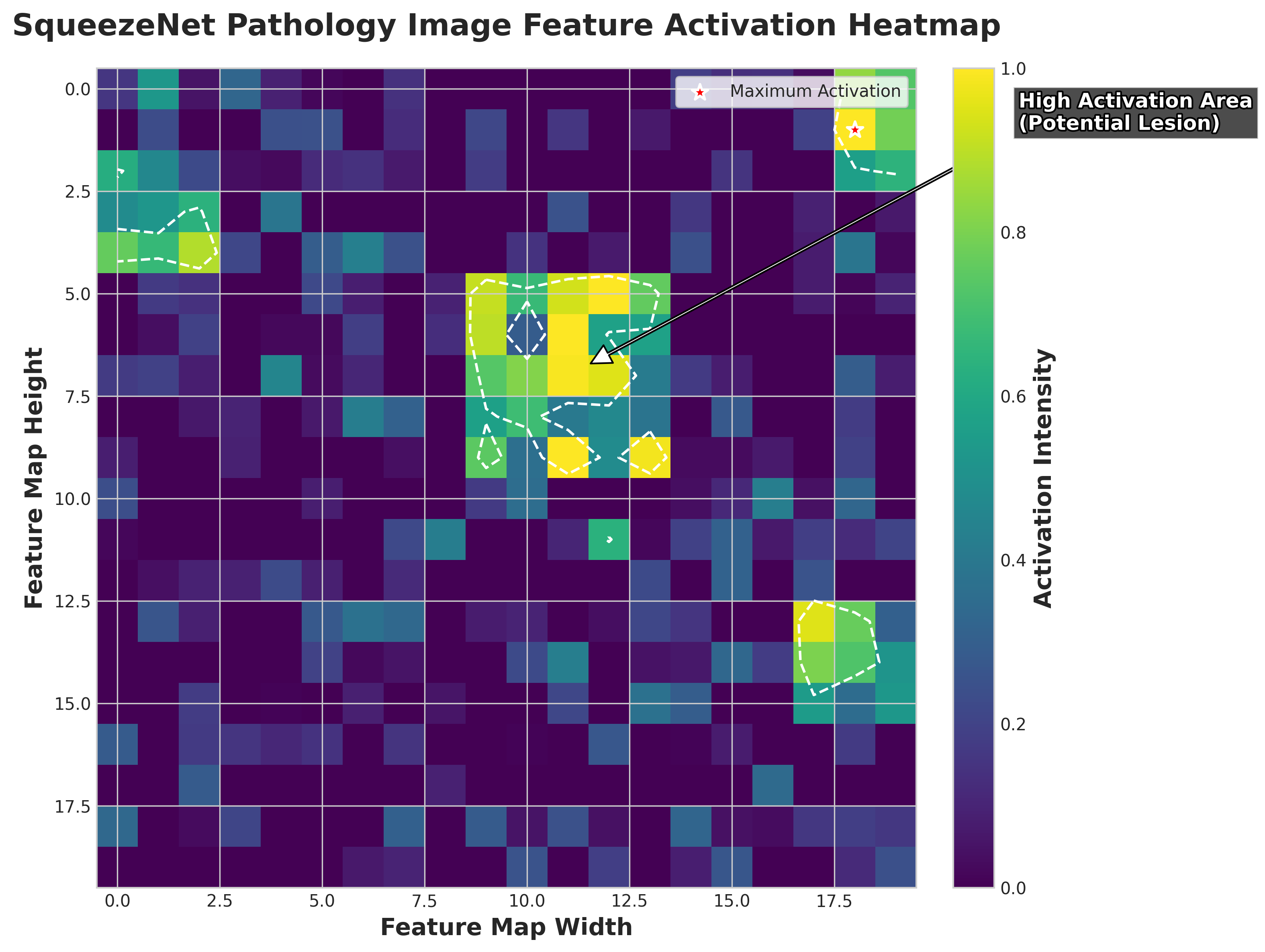


Figure S6-3 Activation heatmap generated by SqueezeNet, visualizing the areas most influential for its classification output.
